# B1-B2 transition in shock-compressed MgO

**DOI:** 10.1126/sciadv.adk0306

**Published:** 2024-06-07

**Authors:** June K. Wicks, Saransh Singh, Marius Millot, Dayne E. Fratanduono, Federica Coppari, Martin G. Gorman, Zixuan Ye, J. Ryan Rygg, Anirudh Hari, Jon H. Eggert, Thomas S. Duffy, Raymond F. Smith

**Affiliations:** ^1^ Dept. of Earth & Planetary Sciences, Johns Hopkins University, Baltimore, MD 21218, USA.; ^2^ Lawrence Livermore National Laboratory, Livermore, CA 94550, USA.; ^3^ Dept. of Earth & Planetary Sciences Div. of Mechanical Engineering, Johns Hopkins University, Baltimore, MD 21218, USA.; ^4^ Laboratory for Laser Energetics, University of Rochester, Rochester, NY 14623, USA.; ^5^ Dept. of Mechanical Engineering and Dept. of Physics and Astronomy, University of Rochester, Rochester, NY 14623, USA.; ^6^ Dept. of Materials Science and Engineering and PULSE Institute, Stanford University, Stanford, CA 94305, USA.; ^7^ SLAC National Accelerator Laboratory, Menlo Park, CA 94025, USA.; ^8^ Dept. of Geosciences, Princeton University, Princeton, NJ 08544, USA.

## Abstract

Magnesium oxide (MgO) is a major component of the Earth’s mantle and is expected to play a similar role in the mantles of large rocky exoplanets. At extreme pressures, MgO transitions from the NaCl *B*1 crystal structure to a CsCl *B*2 structure, which may have implications for exoplanetary deep mantle dynamics. In this study, we constrain the phase diagram of MgO with laser-compression along the shock Hugoniot, with simultaneous measurements of crystal structure, density, pressure, and temperature. We identify the *B*1 to *B*2 phase transition between 397 and 425 gigapascal (around 9700 kelvin), in agreement with recent theory that accounts for phonon anharmonicity. From 425 to 493 gigapascal, we observe a mixed-phase region of B1 and B2 coexistence. The transformation follows the Watanabe-Tokonami-Morimoto mechanism. Our data are consistent with *B*2-liquid coexistence above 500 gigapascal and complete melting at 634 gigapascal. This study bridges the gap between previous theoretical and experimental studies, providing insights into the timescale of this phase transition.

## Teaser

High-pressure properties of an important planetary component, magnesium oxide, are measured under laser shock compression.

## INTRODUCTION

Magnesium oxide (MgO) is a major component of terrestrial planets and has long been a focus of high-pressure research (*1*, *2*). Found on Earth’s surface in small amounts as the mineral periclase, MgO forms a solid solution with FeO and comprises up to 17% of the lower mantle, which in turn accounts for more than half the mass and volume of the planet (*3*, *4*). As the second most abundant mineral in the lower mantle behind the stiffer perovskite-structured silicate (Mg,Fe)SiO_3_, (Mg,Fe)O ferropericlase and its high pressure behavior play an important role in controlling Earth formation and subsequent evolution. (Mg,Fe)O compositions are also expected to be important in many rocky exoplanets (*5*), and the B1-B2 phase transition is expected to occur across a wide range of planet sizes and compositions (*4*).

MgO is studied as a model material for plastic deformation and dislocation mobility over a range of pressures thanks to its simple crystal structure, ionic bonding, and wide stability field (*6*). Like many other binary compounds (*7*), MgO undergoes a reconstructive phase transition from the *NaCl* (*B*1-type, Fm$\bar{3}$ m) to the *CsCl*, (*B*2-type, Pm$\bar{3}$ m) structure with applied/increasing pressure, recently reported between 363 and 580 GPa (*8*–*10*); conditions expected in mantles of rocky exoplanets greater than about five Earth masses in size (*11*). The effect of iron is expected to further reduce the transition pressure (*4*). Empirical systematics and theoretical studies have emphasized the importance of this phase transformation on exoplanetary interior conditions due to an associated strong change in rheological properties with the high-pressure *B*2 phase exhibiting an estimated one hundred times reduction in viscosity (*12*, *13*). Recent theoretical work has found that this *B*1-*B*2 transition boundary (*dT*/*dP*) steepens at high temperatures, as anharmonic effects expand the stability of *B*1-MgO with respect to that of *B*2 (*14*–*16*). Shock compression experiments are ideally suited to test these theoretical predictions as the Hugoniot crosses the *B*1-*B*2 phase boundary at high temperatures [>8000 K, temperatures above which anharmonicity of *B*1 MgO is predicted to greatly increase (*8*, *10*, *14*–*18*)].

Previous indirect measures of this phase transition under shock compression produced conflicting interpretations. Shock velocity studies using electromagnetically driven flyer plates attribute a density excursion on the shock Hugoniot to the *B*1-*B*2 phase transition at 363(6) GPa (where the number in parentheses denotes uncertainty in the last digit) (*10*). Two independent decaying shock studies recorded large temperature excursions along the shock Hugoniot at pressures as low as ∼400 GPa (*8*, *19*), suggesting that the B1→B2 phase transition is accompanied by an unexpectedly large change in enthalpy. Alternatively, this temperature signal may indicate *B*1-MgO melting, a transition not inferred until higher pressures in velocity and reflectivity measurements (500 to 600 GPa) (*8*, *10*, *20*).

Here, we report on experiments that combine laser-driven shock compression with in situ x-ray diffraction (XRD) to reconcile shock velocity, temperature, reflectivity, and ab initio measures of phase transitions along the MgO shock Hugoniot. This configuration allows us to simultaneously measure Hugoniot temperature, crystal structure, and corresponding density at pressures from 400 to 634 GPa (temperatures from 9000 to 14,000 K), constraining the phase diagram of MgO near the B1-B2–liquid triple point.

## RESULTS

Laser-driven shock experiments were conducted on the Omega-EP and Omega-60 lasers at the University of Rochester’s Laboratory for Laser Energetics. Twelve experiments were performed using a shaped laser pulse focused onto the surface of a polyimide ablator to generate shock pressures in the MgO sample ranging from 176 to 634 GPa. XRD (*21*) was used to measure the crystal structure and density of MgO. Peak stress of the MgO was constrained using Doppler velocimetry [VISAR, (*22*)], which monitored the shock-front velocity and reflectivity in a quartz window.

The two lowest pressure shots at 176 and 308 GPa were conducted on polycrystalline MgO. Higher pressure shots were conducted on single-crystal MgO samples with the shock compression direction oriented along the [100] direction [similar to the previous shock experiments of (*8*, *10*, *19*)]. The initially transparent single crystals provided access to determine shock-front temperature using streaked optical pyrometry [SOP, (*23*)], enabling us to measure pressure, temperature, and crystal structure simultaneously.

**Fig. 1.**
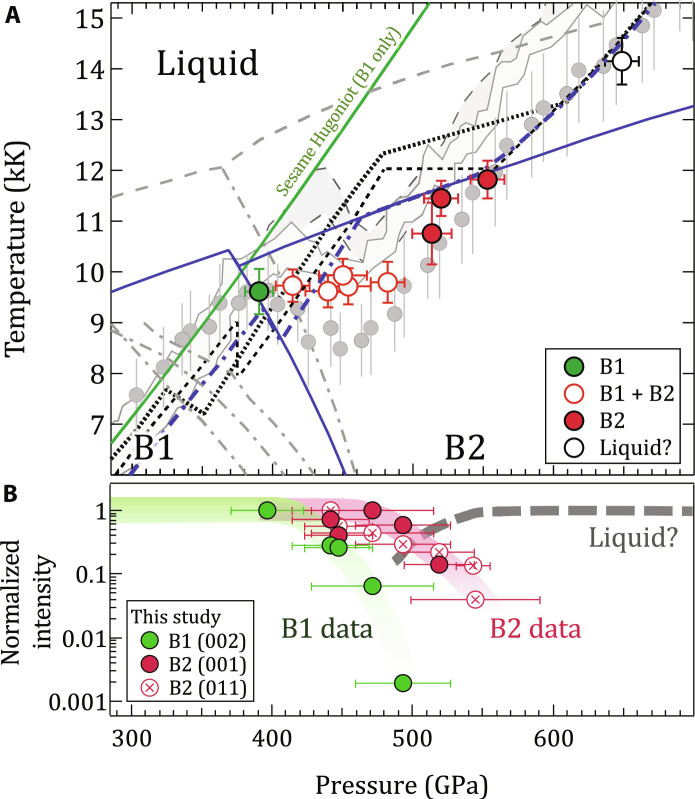
Summary of single-crystal MgO results. (**A**) Measured temperature and phase identifications of MgO as a function of pressure (see also fig. S1). The region of largest disagreement in previous shock experiments [gray bands, (*19*); gray circles, (*8*) with a pressure correction from (*10*); see fig. S11] is between 400 and 500 GPa, which corresponds in this study to that of the mixed B1 + B2 phase region. In contrast, theoretical calculations predict smaller temperature excursions along the shock Hugoniot [dotted (*10*), black dashed (*15*), and blue dash-dot (*16*)]. The corresponding phase boundaries are shown for melting [gray dashed, (*17*)] and the B1-B2 boundary [gray dash-dot, in increasing pressure (*17*, *18*, *77*, *78*)]. B1 and B2 temperatures measured in this study are most consistent with the phase diagram of recent theory [blue solid lines, (*16*)]. The Hugoniot from the single phase (B1-only) Sesame equation-of-state table #7460 is shown as the solid green curve (*64*). (**B**) Integrated diffraction signal for B1 and B2 diffraction peaks as a function of sample pressure (see Materials and Methods). We note that the pressures associated with temperature (top, calculated over a skin depth at the shock front) differ slightly from those from XRD (bottom, calculated over the entire shocked volume). See table S1, and Materials and Methods for details.

Figure 1 shows these results in comparison with recent theoretical calculations and decaying shock experiments. The *B*1→*B*2 phase transformation begins between 397 and 425 GPa and 9610 and 9730 K, consistent with recent quasi-harmonic ab initio molecular dynamic calculations from Soubiran *et al.* (*16*). There is, however, disagreement between previous studies on the Hugoniot temperature in the mixed-phase region (*8*, *19*), indicating that small fluctuations in the sample and loading conditions could manifest in different transition temperatures. In our measurements, we observe a roughly constant temperature across the B1-B2 coexistence region, in contrast with the decaying shock studies that show sharp drops in temperature in this zone. As expected, the phase fraction of B1 versus B2, determined from the XRD signal, decreases with increasing pressure (Fig. 1B).

At higher pressures from 519 to 545 GPa, only the B2 phase is observed. The measurements at 543 and 545 GPa are in agreement with the predicted temperature of a B2 + liquid Hugoniot, and a drop in x-ray signal [including a loss of scattering from B2 (001) lattice planes by 520 GPa] may indicate partial melting, consistent with predictions from theoretical calculations (*10*, *15*, *16*). At our highest pressure of 634 GPa, no solid diffraction is observed. The measured temperatures both below and above the B1-B2 coexistence region are in agreement with some previous decaying shock experiments (*8*).

### Diffraction texture analysis

**Fig. 2.**
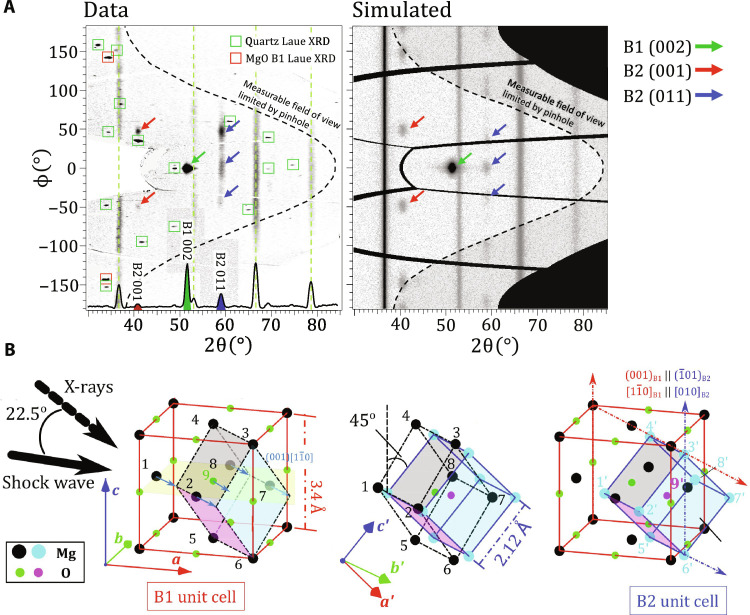
Texture analysis of XRD data. (**A**) Qualitative agreement between experimental image plates projected in 2θ-ϕ and a simulated diffraction pattern at *P* = 442(28) GPa. (Left, bottom axis) An averaged intensity lineout shows features consistent with both the B1 and B2 phases, and Ta calibrant at 1 bar. The red and green boxes represent simulated Laue diffraction locations from MgO B1 and uncompressed quartz [001] single crystals, respectively, from broadband x-ray thermal emission generated within the Cu He-α x-ray source plasma (see Materials and Methods). Also shown are Ta reference peaks (dashed green lines). (**B**) Our data are consistent with the WTM mechanism (*26*, *27*), which is described in two steps: (left) sliding of atoms within alternate (001)_*B*1_ layers in the [1$\bar{1}$0]_*B*1_ direction (blue arrows along yellow plane) to unit cell denoted by black dashed-dot lines, followed by (center) an expansion in the [110]_*B*1_ direction (vectors connecting atoms 1 to 8 and 2 to 7) and a uniform compression perpendicular to this direction, i.e., the [001]_*B*1_ and the [1$\bar{1}$0]_*B*1_ direction (atoms 3 to 6, 4 to 5, 1 to 2, and 8 to 7). The resulting [010]_*B*2_ axis is orientated 45^°^ to [001]_*B*1_. (right) The WTM mechanism results in (001)_*B*1_ || ( $\bar{1}$01)_*B*2_ and [1$\bar{1}$0]_*B*1_ || [010]_*B*2_ and produces six variants that can be detected in our experiments.

Figure 2A (left) shows XRD data from a MgO [100] crystal shock compressed to a pressure (*P*) of 442 ± 28 GPa. The data from five image plate (IP) detectors are combined and projected into 2θ-ϕ (polar) coordinates, where 2θ is the diffraction angle and ϕ is the azimuthal angle around the incident x-ray beam. In these coordinates, diffraction data of polycrystalline material, such as the tantalum pinhole reference, project as straight lines of constant 2θ. The contrasting texture of the B1 and B2 phases of shocked MgO are clearly distinguished from the sharp Laue diffraction of the yet uncompressed quartz single-crystal windows.

The large single-crystal spot at ϕ = 0° from the (002)_*B*1_ lattice plane is consistent with a unimodal orientation distribution centered on the starting orientation of the MgO single crystal with a full width at half maximum of ∼8°. The high pressure B2 phase has a richer texture, which can be used to determine the phase transformation mechanism.

Theoretical calculations have identified two primary energetically favorable mechanisms for the B1-B2 transformation, the Buerger’s mechanism (*24*, *25*), and the Watanabe-Tokonami-Morimoto (WTM) mechanism (*26*). The Buerger’s mechanism involves compression along one of the <111>_*B*1_ directions of the B1 unit cell and an expansion in the B1 orthogonal directions. A proposed modification of the Buerger’s mechanism (*24*)—which introduces a monoclinic distortion to reduce the energy barriers—also produces the same B1-B2 orientation relationship. The WTM mechanism [originally proposed by Hyde and O’Keeffe (*27*)], is depicted in Fig. 2B and is described by a combination of two cooperative motions involving interlayer translation and intralayer rearrangement. Here, sliding of atoms within alternate (001)_*B*1_ layers in the [1$\bar{1}$0]_*B*1_ direction (Fig. 2B, left, blue arrows) is followed by an expansion in the [110]_*B*1_ direction and contraction in all orthogonal directions (Fig. 2B, middle). This results in (001)_*B*1_ || ( $\bar{1}$01)_*B*2_ and [1$\bar{1}$0]_*B*1_ || [010]_*B*2_ (Fig. 2B, right).

Using a forward diffraction model (see Materials and Methods), we find that the Buerger’s transition mechanism leads to a diffraction intensity distribution very different from the one measured in our experiments (see fig. S9B). The WTM mechanism, on the other hand, does lead to an intensity distribution very similar to our measurements (Fig. 2B, right). All six orientation variants of the WTM mechanism were used for this simulation.

Our findings parallel the recent observation of the WTM mechanism for the ∼2 GPa B1→B2 transition in KCl under shock compression (*28*). This mechanism is in contrast to the modified Buerger’s pathway, which is suggested to be favored for the B1-B2 transition of CaO (*29*). The texture analysis indicates that the compressed B1 phase is still highly textured at up to 500 GPa (*V*/*V*_0_ = 2) and 10,000 K (0.9 eV), and transformation into the B2 phase is consistent with a WTM pathway. These measurements up to 634 GPa and 14,150 K provide a direct lattice-level confirmation of the B1→B2 phase transformation on the Hugoniot and the first thermodynamic (*P*-*T*-ρ) constraint of the transformation along any compression path.

### Density determination from XRD

**Fig. 3.**
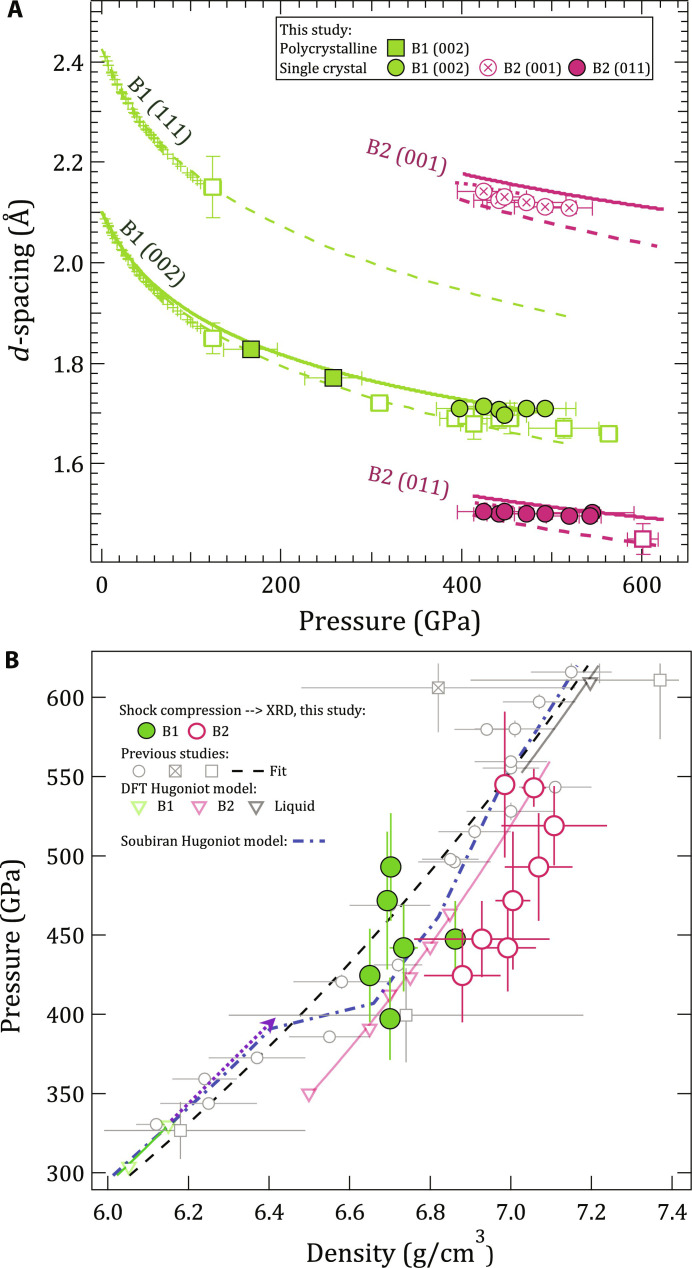
Lattice spacings of observed diffraction peaks of MgO and determined density. (**A**) *d*-spacing as a function of pressure for our data (maroon and green circles) are compared to those measured under ramp compression [open squares, (*9*)]. Static data are shown as crosses with an extrapolation to high pressure using a Vinet EOS fit (green dashed) (*30*). Theoretical *d*-spacing curves for the B2 phase are shown for calculations both at 0 K (*18*) (maroon dashed) and along the Hugoniot (maroon dotted) (*10*). *d*-spacing determined from a cubic fit to Hugoniot shock and particle velocity data are also plotted (solid green and solid maroon curves) (*10*). (**B**) Calculated pressure and measured density for the B1 (green circles) and B2 phases (maroon circles) (see Materials and Methods). Hugoniot data based on shock-speed measurements are shown as the open circle, open square, and crossed square symbols (*2*, *8*, *10*, *58*–*61*). A Hugoniot based on density functional theory calculations is shown as the green (B1), maroon (B2), and black (liquid) open triangles (*10*). Solid line fits to these points are based on linear fits in *U_s_*-*u_p_*. The Hugoniot calculated from quasi-harmonic ab initio molecular dynamics calculations from Soubiran *et al.* (*16*) are shown as the blue dash-dot curve). The purple dashed arrow represents an extension of the B1 phase up to pressure where we see only B1 in our XRD data. An expanded pressure-density plot range is shown in fig. S2.

In situ XRD results are summarized in Fig. 3A, where the lattice spacing of observed *B*1 and *B*2 crystal structures are compared to previous experiments and predictions. The equation of state of single-crystal MgO has previously been measured using a gas-loaded diamond anvil cell [green pulses, (*30*)], and the room temperature isotherm of MgO (green dashed line) was extrapolated from these measurements. Laser-driven ramp compression experiments of polycrystalline MgO (green open squares) followed a compression path closer to the isentrope with a transition to the high-pressure B2 phase at 600 GPa [magenta open square (*9*)].

In this study, we measured the crystal structure of MgO along the Hugoniot. We measured the B1 structure through *d*-spacing measurements of the (002)_*B*1_ plane perpendicular to the shock direction and the B2 structure through the {001}_*B*2_ and {011}_*B*2_ reflections.

Below the phase transition region, the B1 phase is more compressed than expected from previous Hugoniot data, but in the mixed-phase region, in both the B1 and B2 phases, the compression curve appears to flatten out, eventually crossing the fit from previous Hugoniot experiments. This apparent incompressibility of both *B*1 and *B*2 phases is better shown in Fig. 3B, where density is calculated from the *d*-spacing values. Here, *B*2 densities (maroon circles) are an average calculated from an average of the lattice parameters obtained from the (001)_*B*2_ and (011)_*B*2_ reflections. We note that while XRD measures densities for both crystalline phases, the densities reported from Hugoniot *U_s_*-*u_p_* measurements (gray open symbols) represent average values of all phases present.

Our density data, both below and above the mixed-phase region, are in agreement with densities determined by the Root *et al.* (*10*) *U_s_*-*u_p_* study (gray circles). Also similar to the Root data, we observe the Hugoniot points shift to higher density at ∼400 GPa, when compared to an extrapolation of B1-only data (dotted purple line) (*10*). Compared to this extrapolation, we estimate that at 425 GPa, which is our first observation of B2, the volume change due to the B1-B2 phase transformation is 8.6 ± 2.5%. The diffraction data for all shots are shown in fig. S4B along with averaged lineouts of *d*-spacing (fig. S4C). ρ*_MgO_* for each shot is listed in table S1A.

### Optical depth of shock-compressed MgO

**Fig. 4.**
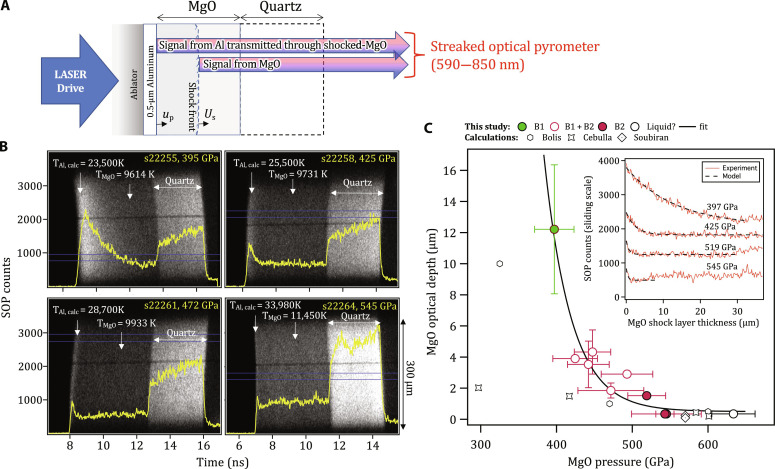
Measurement of optical depth in shocked MgO as a function of pressure. (**A**) The target design consists of a polyimide ablator, a 0.5-μm Al layer directly coated on to an 80-μm-thick MgO [100] single crystal and a 60-μm-thick quartz layer. As the shock propagates through the target assembly, thermal emission from the hot, compressed Al layer, ∼constant over the lifetime of the experiment, is transmitted and attenuated through the shocked-MgO crystal and recorded by the SOP diagnostic. The raw data from the SOP for four different pressures are shown in (**B**) with intensity lineouts, taken from the region defined by two horizontal blue lines, shown as the bold yellow curves. In each case, the calculated Al shock temperature is shown (*79*) as well as the measured MgO shock temperature. (**C**) (Inset) The optical depth is determined by considering the time taken for Al thermal emission to drop from a peak level to 37% of the peak, while the associated MgO shock-thickness is calculated from *U_S_* and *u_P_* estimates. (Main plot) Estimated values of optical depth are plotted along with calculations based on theory (*15*, *19*, *72*) (see Materials and Methods).

The temperature at the shock front during shock transit within the MgO and quartz layers is measured with the Omega-EP SOP (*23*). The SOP records thermal emission integrated over a 590- to 850-nm spectral range, with one-dimensional (1D) spatial resolution over the 300-μm field of view (Fig. 4B and Materials and Methods). The optical depth describes the thickness of shocked material that contributes to the SOP signal. In these experiments, we use the thermal emission from the hot, compressed Al coating as an in situ light source to calculate the optical depth of the shock compressed MgO sample (Fig. 4C). The MgO sample, which is initially transparent, increases in opacity as a function of shock pressure.

Within the B1-only phase at 397 GPa, the shocked MgO is partially transparent, and temperature is effectively measured over an ∼12-μm thickness behind the shock front, evidenced by the slow decrease in measured counts from Al even after the shock wave has entered MgO. At higher pressures, within the mixed B1-B2 phase, temperature is measured over increasingly smaller thicknesses behind the shock front, with an optical depth of <1 μm at 634 GPa. This pressure dependence was fit to the functional form,

$$d=0.5+11.215e^{\frac{-P-397}{35.788}}$$ (1)

where the optical depth *d* is in micrometer and the pressure, *P*, is in gigapascal. Measurement uncertainties account for the variation of thermal emission in time over the 300-μm SOP field of view.

## DISCUSSION

This study provides the first direct experimental constraints on phase relations and structures in the MgO system along the shock Hugoniot. By measuring crystal structure and temperature as a function of pressure, our experiments uniquely bridge previous shock compression experiments on MgO that constrained density and temperature via shock-front velocity (*10*) and pyrometry (*8*, *19*), respectively. X-ray illumination over 2 ns integrates over tens of micrometers behind the shock front, accessing different timescales than those using optical measurements of the shock front. The presented XRD data are, to our knowledge, the highest temperature crystalline diffraction ever reported. It is remarkable that at these extreme *P*-*T* conditions (400 GPa, ∼10,000 K; a ∼1.9× compression), a highly textured B1 microstructure is preserved, with no evidence of large-scale plasticity or grain refinement.

We find that under laser-driven shock conditions, B1-B2 coexistence in MgO spans from 400 to 500 GPa. This large pressure coexistence width is not predicted in any of the theoretical constructions of the MgO phase diagram (Fig. 1 and fig. S1), which could be a result of time dependence in the nucleation of the B2 phase. The constant temperature observed throughout this B1-B2 coexistence region raises the question of whether both the B1 and B2 phases maintain the same temperature or whether it is the average system temperature that remains constant.

Our measurements of B1-only phase up to 397(26) GPa and 9614(450) K, and B2 peaks appearing at 425(30) GPa and 9731(320) K, are in excellent agreement with Soubiran *et al.*’s (*16*) recent quasi-harmonic ab initio calculations of the Hugoniot transition pressures (dashed blue line in Fig. 1A). Earlier theoretical estimates along the Hugoniot predict a lower transition pressure [325 GPa (*10*)] (fig. S1). The progressive decrease of B2 diffraction signal from 519(25) to 545(30) GPa (∼10,700 to 11,450 K) is consistent with B2-liquid coexistence in agreement with previous predictions along the Hugoniot (*10*). Our interpretation of full melt at 634(29) GPa is also consistent with those studies. Future work is needed to provide experimental constraints on the B1-B2 Clapeyron slope and to determine how compression timescale and crystal orientation affect the determined B2 onset pressure.

### Phase transformation kinetics and mixed B1-B2 phase region

The time dependence and onset pressure of phase transitions are affected both by the applied compression rate and the sample pressure relative to the equilibrium phase boundary (*31*, *32*). Therefore, a consideration of strain rate effects is important when relating observations under dynamic compression with those from equilibrium models. On a macroscopic scale, classical nucleation theory describes the dynamics of phase transitions through a two-stage process: initial slow transformation (nucleation) followed by a rapid growth regime in which the transforming interface can flow with velocities approaching the local speed of sound (*32*, *33*). These processes are consistent with atomistic simulations of the B1→B2 phase transformation (*34*, *35*).

Under shock compression, if the phase transformation kinetics are much faster than the experimental measurement, then an equilibrium state can be accessed. If, however, phase transformation kinetics are comparable to the experimental measurement lifetime, then the onset pressure of the transition will deviate from equilibrium values. In most reported studies, kinetics result in phase transitions being observed at higher pressures compared to the equilibrium phase boundaries (*31*, *32*).

**Fig. 5.**
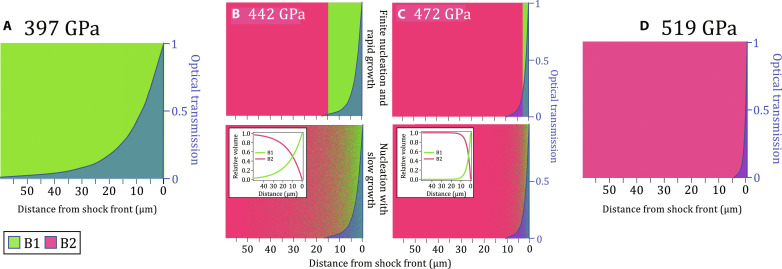
Possible distributions of phase fractions within compressed MgO. (**A**) Representation of a compressed volume of MgO at a shock pressure of 397 GPa (B1-only, green). On the basis of the measured optical depth at this pressure (∼12 μm, Fig. 4C), the optical transmission as a function of distance away from the shock front can be calculated (blue-shaded region). This represents the relative volume-dependent contribution to the SOP temperature measurement (as plotted in Fig. 1A). (**B** and **C**) represent pressures within the XRD measured mixed B1 + B2 phase region (Fig. 1A). For each pressure, the relative proportion of B1 (green) and B2 (maroon) phases are estimated by relative changes in normalized XRD intensity with pressure, as plotted in Fig. 1B. As our XRD data are volume-integrated, we cannot determine how the B1 and B2 phases are distributed within the compressed MgO, and knowledge of this distribution within the optical skin depth is needed to correctly interpret the measurements of temperature. Here, we consider two possibilities based on different transformation kinetics models: (top) finite nucleation and rapid (instantaneous) growth into the B2 phase, which results in a two-phase structure with distinctly separated B1 and B2 volumes and (bottom) nucleation with slow growth, resulting in a random mixed-phase assemblage within the measured optical skin depth. Our data are most consistent with the latter model and B2 nucleation time scales of <0.25 ns. The representation in (**D**) for a shock pressure of 519 GPa is B2-only (maroon) with a measured optical depth of <1 μm (Fig. 4C).

Our combined temperature, diffraction, and optical depth data allow us to place constraints on the timescale of the B1-B2 transition. Within our data, the observed shock temperature of the mixed B1 + B2 phase region in Fig. 1A remains constant from ∼420 to 490 GPa. This pressure range is several times larger than equilibrium calculations of mixed phase along the B1-B2 phase boundary (fig. S1). In our experiments, density is determined from diffraction collected over the whole sample depth, while temperature is measured only from the front portion of the compressed volume, depending on the optical depth. Therefore, XRD cannot distinguish between a B1-B2 mixture or distinctly separated B1 and B2 volumes (Fig. 5). Observation of a constant temperature in the mixed-phase region, rather than a continual increase with pressure along the B1 Hugoniot (green curve in Fig. 1A), indicates that the SOP samples both the B1 and B2 phases and is therefore consistent with the existence of a mixed-phase assemblage within the optical skin depth. Considering a shock velocity of 16.3 μm/ns at 442 GPa (*10*), this places an upper limit of ∼0.25 ns on nucleation into the B2 phase at this pressure. This is much faster than the sample compression timescale of ∼3 to 4 ns.

As observed in other phase-transforming materials (*31*, *32*), the time required for nucleation and growth into the new phase is inversely proportional to the excess pressure above the equilibrium transformation pressure (*32*). Therefore, with increasing pressure, the B1 fraction within a shocked volume is expected to diminish (as observed in Fig. 1B).

The highest pressure we observe only compressed B1 is 397(26) GPa. The shock transit time through the MgO sample at the time of x-ray exposure was ∼3.25 ns. We note that this pressure may lie within the B2 stability field if the transformation time into the B2 phase is slower than this shock transit time. The lowest pressure we observed the B2 phase is 425(30) GPa. Because of uncertainties in the phase transition kinetics, we consider this to be an upper limit on the *B*1-*B*2 onset pressure. This upper limit is lower than the most recent theoretical predictions by Zhang *et al.* (see fig. S1) (*36*).

### Anomalous density of B1 phase

In our experimental geometry, measurements of the (002)_*B*1_ reflection provide a constraint of the lattice parameter in the direction perpendicular to the shock propagation direction. Only a single reflection was detected because of the highly oriented nature of the *B*1 crystal and the XRD geometry. In this uniaxial compression geometry, and in the presence of deviatoric stresses (not constrained here), densities calculated from this reflection are likely an underestimate. This is in contrast with experiments that measure the Hugoniot through *U_S_* -*u_P_* measurements along the shock propagation direction, in which the calculated densities may be an overestimate (*10*).

In the previous *U_S_* -*u_P_* measurements of MgO, a density excursion away from the B1 Hugoniot starting at 363 GPa suggested a phase transition to the denser B2 phase (unfilled circles in Fig. 3B) (*10*). In this study, we also observe the density excursion at 397 GPa at a density of 6.746 g/cm^3^, but MgO remains in the B1 phase (Fig. 3B). This suggests elastic softening caused by phonon anharmonicity preceding the phase transformation. Anharmonicity in MgO is predicted to reduce both thermal expansion with increasing pressure and bulk modulus with increasing temperature (*37*). In both cases, this would serve to increase compressibility. In addition, theoretical studies on MgO show evidence of C_44_ elastic constant softening within the B1 and B2 phases at pressures approaching the phase transition (*38*).

At higher pressures where the B2 phase appears, the density of the B1 phase fraction deviates from both the B1 and B2 Hugoniot curves and remains nearly constant from 397 GPa all the way to 493 GPa. However, the overall density of the material does increase with increasing pressure, since at higher pressures, a larger fraction of the denser B2 phase is present. Whereas the B1 phase is stable or metastable below ∼6.75 g/cm^3^, when compressed above this density, it becomes unstable and spontaneously transforms into the B2 phase. This behavior is not uncommon and has been observed in multiple other laser-shock studies (*39*, *40*).

Another potential explanation could be that pressure gradients in the sample cause regions compressed to higher pressure to transform to the B2 phase, while lower-pressure regions remain as B1. However, the measurement of constant temperature through the mixed-phase region shows that both phases are present within the optical skin depth at the shock front, suggesting against this possibility (see section below for more details).

### Effect of optical depth on temperature determination

Figure 4C shows the experimentally determined optical skin depth as a function of shock pressure. For the SOP determination of shock temperature (Fig. 1A), thermal emission is collected from an extended volume that encompasses the shock front and pressure states behind the shock front. This volume is defined by the optical depth. The values reported in Fig. 4C are substantially higher than values assumed in shock decay studies of MgO (*8*, *19*). For example, in the study of McWilliams *et al.* (*8*), the optical depth of shocked-MgO was taken as ∼1 μm (or negligible) for pressures above 300 GPa. For those experiments, and because of strong pressure and temperature gradients behind the shock front, a large optical depth in the B1 phase will give rise to an overestimation of shock-front temperature (*8*), which will diminish as a function of increasing pressure. A detailed correction of previously reported shock decay data based on the optical depth values in Fig. 4C is beyond the scope of this paper.

In temporally steady shock compression experiments, as reported here, there are minimal pressure gradients behind the shock front, and the determination of temperature is unaffected by a pressure-dependent optical depth as long as the shocked thickness exceeds the optical depth, e.g., Fig. 5 (A and D). However, as discussed above and illustrated in Fig. 5 (B and C), complications in interpreting SOP data arise for mixed-phase volumes.

We note that molecular dynamics simulation studies of shock compression in materials with minimal plasticity have reported that shear relaxation can result in the formation of supercooled liquid states within a tens-of-nanometer–thick layer behind the shock front (*41*, *42*). While we have no direct evidence that these states exist in our shock-compressed MgO samples, their presence could potentially modify the measured temperature.

## MATERIALS AND METHODS

### Sample preparation 

The target design for experiments on Omega-EP is shown in Fig. 4A and consists of a 125-μm-thick polyimide (C_22_H_10_N_2_O_5_) ablator, 75-μm-thick single-crystal MgO [100], and 50-μm-thick z-cut single-crystal quartz (α-SiO_2_) assembled with ∼1-μm-thick glue bonds. The high-purity MgO single crystals (>99.95% purity, <100> ± 0.5°, density 3.58 g/cm^3^) were supplied by MTI corporation. Typical impurities are <50 parts per million. A 0.5-μm-thick Al layer was deposited directly onto the MgO crystal to enhance reflectivity for velocimetry measurements, and an antireflection coating was applied to the quartz-free surface to suppress photon back reflection at quartz/vacuum interface. The target design for a subset of shots on the Omega-60 laser on polycrystalline MgO [99.5% purity, further described in (*43*)], consists of a 100-μm-thick polyimide layer, 50-μm-thick polycrystalline MgO, and a 50-μm-thick quartz layer.

### Laser configuration

On Omega-EP, a 10-ns 351-nm laser pulse was focused to a 1.1-mm diameter spot on the polyimide front surface. For the experiments on Omega-60, an ∼7.4-ns composite pulse shape was built with two 3.7-ns laser pulses focused to a 0.8-mm diameter spot. Spatial smoothing of the focal spot intensities was achieved with distributed phase plates inserted into the beamlines. In both cases, laser ablation resulted in uniaxial compression of the target assembly in a near temporally steady shock. By varying the laser intensity, the pressure in the MgO sample was systematically increased from 176 to 634 GPa. In total, 12 shots were performed [10× [100] single crystal (Omega-EP), 2× polycrystalline (Omega-60)] (see fig. S3).

### XRD measurements

A laser-generated Cu plasma illuminated the shocked MgO, at an incidence angle of 22.5° to target normal, with quasi-monochromatic x-rays (8.37 keV, 1.48 Å) for 2 ns (*44*). Diffracted x-rays, collimated by a Ta pinhole positioned directly behind the target are recorded in transmission geometry on Fujifilm BAS-MS IP detectors (*21*, *45*). The IP’s store incident x-ray energy in phosphor elements that are then read into units of photostimulated luminescence using a calibrated scanner. IPs have broad spectral sensitivity, a linear response to x-ray fluence, and a high dynamic range (>10^5^). Diffraction peaks from the uncompressed Ta pinhole are used to accurately determine the experimental geometry, i.e., the position of the x-ray source and scattering center within the sample, with respect to the various IP detector panels. We also perform 2D statistics-sensitive nonlinear iterative peak-clipping background subtraction and angular-dependent corrections as in (*21*, *46*). The IP planes are projected into 2θ (diffraction angle) - ϕ (azimuthal angle) space in Fig. 2A, or in ϕ - *d*-spacing coordinates as shown in fig. S4B.

#### Normalized diffraction intensity

The shot-to-shot diffraction intensity is normalized in Fig. 1B by accounting for variations in the x-ray source flux and any variations in the compressed sample volume. The former is constrained by considering the intensity of the uncompressed Ta pinhole peaks, and the latter is determined from the VISAR record. In addition, a background subtraction is performed by subtracting the signal from adjacent regions on the IP where no crystalline diffraction is observed.

### Forward model for XRD texture analysis

Forward diffraction simulations were performed to predict the expected signal for an arbitrary crystal orientation distribution (e.g., Fig. 2A and fig. S9B). Given all experiment parameters, the model computes the expected intensity distribution in 2θ − ϕ space. The parameters include the crystal structure, lattice parameters, phase fractions, and crystallographic texture of compressed and high-pressure phases. The texture information is used to modulate the powder diffraction intensity in the azimuthal direction. In addition to the material-related parameters, the model also takes the peak shape functions as inputs. These parameters can be used to specify the instrumental broadening, microstrain, and grain size effects. The crystallographic texture is represented using a finite element representation of the Rodrigues space fundamental zone (*47*–*49*). The forward model calculation is done in two steps: (i) The powder diffraction intensity for the B1 phase, B2 phase, and Ta pinhole is calculated as a function of 2θ. Since this is a powder diffraction calculation, the intensity along the ϕ dimension remains constant. (ii) The assumed unimodal orientation distribution function for the starting B1 phase and the predicted B2 phase orientation from WTM/Buerger’s mechanism is projected as pole figures (figs. S6, S7, and S8). This gives us the intensity variation along the azimuth for the B1 and B2 phases, which is multiplied with the powder intensity distribution from step (i). The area masked by the pinhole is superimposed on the intensity distribution obtained from these calculations. Note that the absorption due to varying x-ray path length after diffraction is not accounted for.

#### Consistency with the WTM mechanism

To test consistency with previously reported values of stretches required in the phase transition, our XRD-determined lattice parameters were used to compute the principal stretches (i.e., expansions/contractions) for both mechanisms. The stretches for both mechanisms are reasonable and only differ by a few percent from previously reported values for NaCl (see sections S1 and S2 and fig. S10) (*50*). However, using the forward diffraction model, we find that the Buerger’s transition mechanism leads to a diffraction intensity distribution very different from the one measured in our experiments (see fig. S9). We therefore consider that this mechanism is not active. On the other hand, the orientation relationship predicted by the WTM mechanism leads to diffraction signal reflections consistent with our data (Fig. 2A). While at a pressure of 442 GPa, which is just above the B1-B2 phase boundary, the azimuthal texture is clearly consistent with the WTM phase transformation mechanism (Fig. 2A), at higher pressures the texture becomes more refined, possibly due to grain rotation/plastic deformation within the B2 phase (fig. S4B).

#### Mosaic spread

To get good qualitative agreement with our XRD data (Fig. 2A), we find that it is necessary to introduce a level of mosaic spread to the B1 and B2 phases (∼8° and 10°, respectively). This is akin to using a unimodal orientation distribution function for both the phases. The mosaic spread is a measure of the orientational order of the crystallites comprising the bulk material: The smaller the mosaic spread, the greater is the orientational order of the sample (*51*, *52*). The mosaic spread is defined as full width at half maximum of the orientation distribution function. The larger the mosaic spread, the greater is the crystal rocking curve and the more likely Bragg diffraction conditions are met. The orientation of compressed B1 phase centered on the orientation of the ambient B1 phase, whereas the orientation of the B2 phase is centered on the six orientations generated by the WTM mechanism.

### Shock velocity measurements

**Fig. 6.**
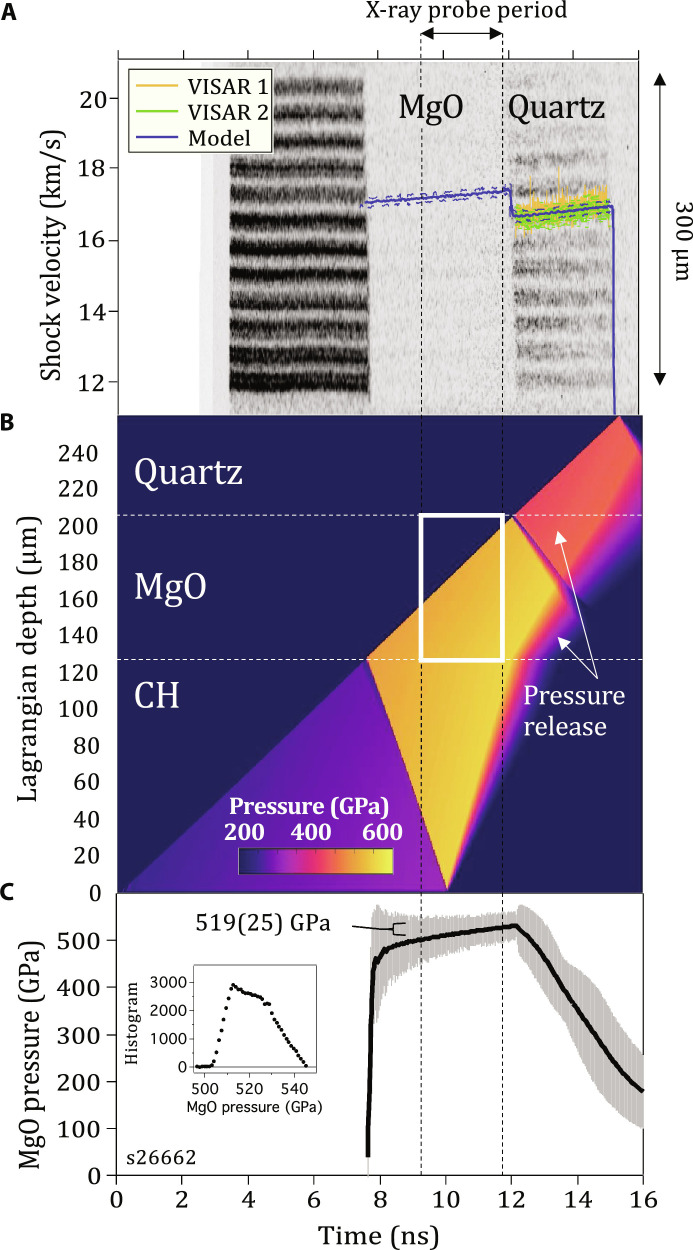
Pressure determination of shock compressed MgO. (**A**) Raw VISAR image with shock transit periods within the MgO and quartz layers highlighted. Shock velocity in quartz, *U_S_*,*_Qtz_*(t), measured by two independent VISAR channels, is shown with uncertainties as the orange and green traces. Simulated *U_S_* (t) through the MgO and quartz layers are shown as the blue trace, with dashed blue error bands based on experimental measurement uncertainties. Time = 0 ns represents the laser turn on time. (**B**) *P*(x,t) output from an HYADES hydrocode simulation (*57*). (**C**) Calculated average pressure versus time within the MgO sample with uncertainties that reflect the pressure distribution during the x-ray probe period. Inset figure shows a histogram of the pressure states within the MgO sample during the x-ray probe time [white box in (B)]. See Materials and Methods for details.

Shock velocity is measured using a line-imaging VISAR with two independent channels set with different velocity sensitivities (4.3697 or 7.8167 and 3.1903 km/s per fringe shift for VISAR channels 1 and 2, respectively) to provide independent measurements of velocity and resolve any ambiguities associated with sharp jumps that exceed the time response of the system (*53*). We use standard ambient pressure values for c-cut quartz (α-SiO_2_): ρ_0_ = 2.648 g/cm^3^, and a refractive index at the 532-nm VISAR wavelength of 1.547. A representative VISAR interferogram, which encodes velocity information as fringe shifts, is shown in Fig. 6A.

At early times, the 532-nm VISAR probe beam reflects off a 0.5-μm-thick Al layer, which is coated directly onto the MgO surface facing the polyimide. As the shock enters the 75-μm-thick MgO layer (at ∼7.8 ns), the target reflectivity dissipates (no fringes) because of the combined effects of shocked-region opaqueness, nonreflectivity of the shock front (*8*, *19*), and a loss in aluminum reflectivity as a function of shock temperature (*54*). After transit through MgO, the shock enters the 50-μm-thick quartz layer (∼12 ns). For shock states greater than 100 GPa, shocks in quartz are reflective, and, under these conditions, VISAR records quartz shock velocities directly (*55*). *U*_*S*,*Qtz*_(*t*) is measured with the two VISAR channels (orange and green curves with 1σ uncertainties). Each velocity measurement is determined from the average and SD over a 300-μm field of view, which captures any increased velocity distribution due to spatial nonplanarities within the drive (e.g., shock-front tilt due to gradients in the glue layer thickness). In addition, we use a conservative estimate in velocity uncertainty based on an assumed 5% accuracy in determining fringe phase shift. A summary of the laser pulse shapes used and the associated measured *U_S_* (t) traces for all shots are shown in fig. S3.

### Pressure determination

Over the pressure range of our study, the shock front in MgO is not highly reflective (*8*), while the shock front in quartz exhibits metallic-like reflectivity (*56*). Using VISAR, we measure the MgO shock entry and exit times and the quartz shock velocity *U*_*S*,*Qtz*_(t). The pressure history in MgO is determined by simulating the experimental conditions with a 1D hydrocode, HYADES (*57*), which calculates the hydrodynamic flow of pressure waves through the target assembly in time (*t*) and space (*x*) (Fig. 6B). The inputs to the hydrocode are the thicknesses of each of the constituent layers of the target, including the measured epoxy layer thicknesses (∼1 to 3 μm), an equation-of-state (EOS) description of each of the materials within the target, and laser intensity as a function of time, *I_Laser_*(*t*). We find that pressure (gigapascal) in the polyimide ablator scales as 4.65 × *I_Laser_*^0.8^, with *I_Laser_*(*t*) (PW/m^2^) calculated from measurements of laser power (fig. S3A) divided by an estimate of the laser spot size. The latter is not well defined, however, and so a scaling factor is applied to *I_Laser_*(*t*) to obtain improved agreement with the experimental observables.

A series of forward calculations were run with iterative adjustments of *I_Laser_*(*t*) (few % level) until convergence was reached between the calculated *U*_*S*,*Qtz*_(*t*) and the average of the measured *U*_*S*,*Qtz*_(t) curves (solid blue curve in Fig. 6A). Once achieved, the calculated *P*(*x*,*t*) (Fig. 6B) was used to determine *P*_MgO_ ± *P*_Distribution_ during the x-ray probe period (white box in Fig. 6B) from the pressure distribution histogram peak (*P*_MgO_) and full width at half maximum (*P*_Distribution_) (e.g., inset to Fig. 6C). The steps described above were repeated to match the bounds of *U*_*S*,*Qtz*_(*t*) experimental uncertainty (dashed blue curves in Fig. 6B). In this way, the final determined calculation of *P*_MgO_ ± *P*_Distribution_ was directly related to the experimental uncertainties. This method of pressure determination explicitly accounts for any temporal non-steadiness in the compression wave. The HYADES fits to the experimentally determined *U*_*S*,*Qtz*_(t) curves for all shots are shown in fig. S3B. Our experimental geometry also permits temporal measurements of MgO shock entry and exit times, which permits calculation of average *U_s_*, but does not capture deviations in *U_s_* due to non-steadiness of the drive. Nevertheless, these transit time *U_s_* values gave sample pressures (*10*) in general consistency with the approach outlined above.

Any tilt in the drive due to, for example, a nonuniform thickness in the glue layer between the polyimide and MgO, will cause the shock in one part of the MgO to be delayed relative to the other part of the MgO (e.g., Fig. 6). In steady shock experiments, this does not necessarily result in an additional pressure distribution during the x-ray probe time. Nevertheless, in our analysis approach, the distribution of velocity over the VISAR field of view is explicitly considered in the pressure determination and the reported uncertainty in the pressure.

We note that the XRD determination of structure is volume-integrated, whereas the temperature is measured over an optical skin depth at the shock front. Therefore, the pressure associated with XRD and *T* measurements are calculated accordingly (as reported in table S1). For this reason, the pressures for the data in Fig. 1 (A and B) are slightly different.

In our experiments, the sample is uniaxially compressed. While the use of the term “pressure” throughout the paper suggests a hydrostatically compressed state, we cannot rule out the presence of deviatoric stresses, which would, in the case of our measurements and all previous Hugoniot measurements (*2*, *8*, *10*, *58*–*61*), give rise to higher values of longitudinal stress and therefore reported pressure. In the analysis of Fowles (*62*) using the Levy–von Mises yield criterion (*63*), this stress deviation corresponds´ to two-thirds the yield strength. However, the high-pressure strength of MgO is unknown.

#### EOS tables

The EOS tables used in the hydrocode simulations for MgO (Sesame #7460) and quartz (Sesame #90010) describe Hugoniot pressure–particle velocity paths as shown in fig. S5A (*64*). Also plotted are Hugoniot data for MgO (*8*, *10*, *59*, *60*) and quartz (*65*). Pressure residuals between measured Hugoniot data and the calculated Hugoniots are shown in fig. S5B. While Sesame #7460 is in good agreement with previous data on MgO, there is an average systematic offset of 1.6 ± 1.9 GPa between the quartz Hugoniot data and the quartz EOS table used in the pressure determination calculation over the pressure range of our study. This level of disagreement produces a systematic offset in the calculated MgO pressure but at a level much less that other contributors to pressure uncertainty, e.g., the pressure distribution within the sample due to temporal non-steadiness of the shock drive (Fig. 6C).

#### Uncertainty in pressure

The MgO EOS used for pressure determination does not describe the expected ∼5% volume collapse associated with the B1→B2 phase transformation. This will introduce a systematic offset in the determined pressure for pressures above the B2-phase onset. However, this offset is expected to be small relative to the calculated pressure distribution and is thus neglected. Additional contributions to pressure uncertainty that are small relative to the calculated pressure distribution are uncertainties in laser beam timing (50 ps), VISAR timing (50 ps), sample thicknesses (1 μm), quartz refractive index uncertainty, and the deviations between the hydrocode-calculated *U*_*S*,*Qtz*_(*t*) and the experimentally determined *U*_*S*,*Qtz*_(*t*) (Fig. 6A). Calculated *P*_MgO_ ± *P*_Distribution_ for all shots are listed in table S1A.

#### Pressure uniformity

In our analysis, we assume that there is a uniform pressure state between the B1 and B2 phases. For homogeneous nucleation of the B2 phase within the B1 lattice (*35*, *66*), this is a valid assumption as, in shock experiments, pressure is continuous across interfaces (e.g., Fig. 6). We also considered other sources that would potentially lead to pressure distributions within our sample. For example, in many phase-transforming systems under shock compression, a two-wave structure can form (*67*). The first and faster-moving wave is pinned at the pressure of the phase boundary, and this is followed by a second slower-moving phase-transforming wave. At higher compression, the phase-transforming shock eventually overtakes the initial phase boundary wave to produce a single shock front (*67*). This would pin the B1 phase fraction within the B1 + B2 mixed phase at a common pressure of 397 GPa, while the B2 phase fraction would be at the phase-transforming wave pressure. We considered this possibility in our experiments as it would be consistent with our B1 density data in which the density remains roughly constant over the mixed-phase region.

However, we ruled out this interpretation based on two arguments: (i) In a multiwave system, both waves will transmit into the quartz layer, but only the first shock will be detected through optical velocimetry because that initial shock has a reflecting shock front (and therefore the second phase transformation shock is not visible). In this multiwave picture, increasing laser energy would not result in a detectable increase in the first shock *U_s_* as this shock should be pinned at the phase boundary pressure, but this is not what is observed experimentally. (ii) In a multiwave system, the first wave would contain only the compressed B1 phase. However, our temperature measurements over ∼2- to 4-μm skin depth behind the shock front indicate that this volume represents a mixed-phase assemblage (see Fig. 5 and associated discussion).

Other factors that potentially could introduce gradients in our sample are laser speckle imprint from the drive laser (*68*) and x-ray heating (*69*). For the former, the analysis of Gorman *et al.* (*68*) suggests that there is sufficient smoothing over the 125-μm polyimide thickness to anneal out any high-frequency laser speckle imprint. X-ray emission from the laser plasma—expected in the 1- to 3-keV range—is efficiently absorbed by the polyimide; however, there may be some degree of concurrent heating from the x-ray source used for the XRD. This would tend to preferentially heat the MgO volume closest to the drive surface (*69*). Previous estimates of this effect on more efficient x-ray absorbers than MgO have suggested that concurrent heating of ∼400 K is possible (*69*).

### Optical skin depth

In Fig. 4C, the experimentally determined optical skin depth as a function of shock pressure is shown. These values are determined by considering the time taken for the recorded Al thermal emission to drop from a peak level to 37% of the peak (1/e) while calculating the shock-thickness over this period from estimates of MgO *U_S_* and Al/MgO *u_P_*—as determined from hydrocode simulations of the experimental conditions (e.g., Fig. 6A). These simulations also confirm that the Al temperature is ∼constant over the lifetime of the experiment. As thermal conductivity is poorly constrained at our compression conditions, in our hydrocode simulations, we used fixed values determined at ambient pressure conditions. The corrected SOP traces exhibit a clear Beer-Lambert behavior, and exponential fits yield values for optical depth d = 1/α, where α is the absorption coefficient (see inset to Fig. 4C).

While, for convenience, we assume a constant Al temperature over the lifetime of the measurement, this is a good assumption for the following reasons. To focus on one example, we consider shot s22261 (472 GPa) in Fig. 6B. Here, the temporal information needed to determine the optical depth is related to how rapidly the hot thermal emission from the Al decays down to the baseline emission set by the MgO shock temperature. For s22261, this happens within ∼500 ps. Next, for the analysis, we investigated whether or not that exponential decay in Al thermal emission could be related to (i) temporal features in the drive and/or (ii) thermal transport from the Al layer to the surrounding layers. We rule out (i) based on hydrocode simulations and our understanding of the temporal structure of the laser shock.

For (ii), we note that the Al layer is bounded by an epoxy layer on the laser drive side and MgO on the other side. For the example shot s22261, the question is whether the shock temperature in Al set up by the initial shock is modified over ∼500 ps because of heat flow into/from the surrounding layers. This is governed by the shock temperatures within the epoxy, Al and MgO layers, and the thermal conductivities of the respective layers. As the pressure dependence of the thermal conductivity (κ) is not well constrained, we assume in our HYADES code simulations that the κ values are fixed at the ambient values. These values are used for every layer in the target assembly, and specifically for the layers of interest, we have: epoxy (1.02), Al (237), MgO (*42*), where the values in parentheses are reported ambient κ’s in units of W/m.K. We note that recent theoretical predictions suggest that the large difference in ambient κ between Al and MgO will increase with pressure due to a three- to four-times drop in MgO κ across the B1-B2 phase boundary (*70*) and an increase in Al κ (*71*). In our experiments, the initial shock temperature in the aluminum layer is substantially higher than those in the surrounding layers. This temperature in the code does not change significantly because the surrounding layers are insulating (lower κ), and so there is limited heat flow, with time, from the Al into the epoxy and MgO layers. For this reason, the assumption of a constant temperature state in the Al, which we use as a simplifying assumption in our analysis, is a good one.

Calculations of optical depth along the Hugoniot are also shown from Bolis *et al.* (*19*) in Fig. 4C. The optical depth is also determined from the shocked-MgO conductivities calculated by Cebulla *et al.* (*72*) and Soubiran *et al.* (*15*) using the expression (*73*)

$$d\left(E\right)=\frac{\epsilon_{0}\mathrm{cn}\left(E\right)}{\sigma \left(E\right)}$$ (2)

where *E* is the photon energy (centered on 2 eV for SOP), *c* is the speed of light, ϵ_0_ is the vacuum dielectric permittivity, and σ is the calculated shocked MgO electrical conductivity (*15*, *72*). The refractive index, *n*, for shocked MgO is estimated through extrapolation of the dependency reported in (*60*).

### Temperature determination

**Fig. 7.**
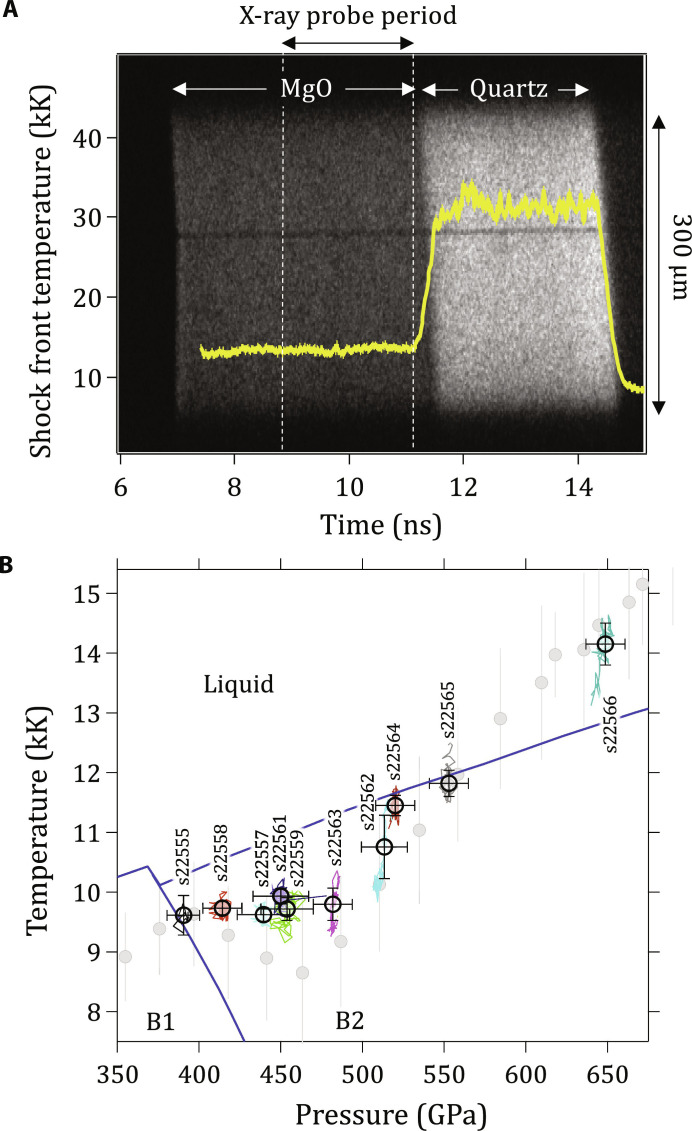
Determination of MgO temperature from pyrometry measurements. (**A**) Raw SOP data for shot s22264 [*P* = 520(12) GPa], where thermal emission from the MgO and quartz layers, over the 300-μm field of view, are indicated. Also plotted is the calculated shock-front temperature (yellow; see Materials and Methods). (**B**) Measured MgO shock-front temperature plotted as a function of calculated MgO shock-front pressure during the x-ray probe period. The shot number for each data point is shown. Recent quasi-anharmonic calculations by Soubiran *et al.* (2020) for the melt, and the B1-B2 phase boundary (blue curves) are also shown (*16*). The gray circles represent decaying shock measurements by McWilliams *et al.* (*8*), which have been corrected in pressure based on the subsequent *U_s_*-*u_p_* measurements by Root *et al.* (see fig. S11) (*10*). Data points (as plotted in Fig. 1A) are shown as circles with uncertainties that represent the SD of the measured temperature and calculated pressure distribution (colored curves) during the probe period. An additional estimated ±300 K random temperature uncertainty associated with SOP measurements is combined with the distribution error bars shown here for the uncertainties shown in Fig. 1A (see Materials and Methods).

The temperature at the shock front is measured with the Omega-EP SOP (*23*). The SOP records thermal emission integrated over a 590- to 850-nm spectral range and spatially resolved over the 300-μm field of view (e.g. Fig. 7A). Temperature is calculated assuming gray-body emission where emissivity, ϵ, is defined as ϵ = 1-*R*, where *R* is sample reflectivity. Temperature as a function of time is given by

$$T\left(t\right)=\frac{T_{0}}{\text{ln}\left\{1+\frac{\left[1-R\left(t\right)\right]A}{I\left(t\right)}\right\}}$$ (3)

where *T*_0_ and *A* are calibration parameters related to the SOP setup (*23*), *I*(*t*) is the measured intensity of shock-front thermal emission, and *R*(*t*) is measured at the 532-nm VISAR wavelength. As *T*_0_ essentially captures the spectral response of the SOP system, and T depends only weakly on this parameter, we assume that it is constant: *T*_0_ = 1.909 eV (*23*, *74*). As potential vignetting through the SOP collection and transport optics can affect the intensity of thermal emission collected, the measured properties of the quartz sample are used as a standard to calibrate the SOP, i.e., to determine the value of *A* for each shot (*56*). The relationship between shock velocity, shock-front temperature, and reflectivity in quartz are well known over the pressure range of our experiments (*56*, *75*). On the basis of these previous studies, we use the following relationships (*56*)

$$R_{\mathrm{Qtz}}=4.614\times 10^{-3}+\frac{\left(0.3073-4.614\times 10^{-3}\right)\times U_{S,\mathrm{Qtz}}^{9.73}}{U_{S,\mathrm{Qtz}}^{9.73}+16.185^{9.73}}$$ (4)

and

$$T\left(K\right)=1421.9+4.3185\times U_{S,\mathrm{Qtz}}^{2.9768}$$ (5)

where *R_Qtz_* is the quartz reflectivity as measured at the VISAR probe wavelength of 532 nm. As we measure *R_Qtz_* and *U*_*S*,*Qtz*_, this enables us to determine the constant *A* in Eq. 3. SOP data for a representative shot are shown in Fig. 7A, where thermal emission from the MgO and quartz layers and the x-ray probe period are indicated. Also plotted is the calculated shock-front temperature as a function of time (yellow). Our analysis approach assumes radiation and material thermal equilibrium. This is true for optically thick systems and is valid in our experiments as the shocked MgO thickness exceeds the determined optical skin depth (see Fig. 4 and associated discussion). For each time step, temperature is measured at the shock front, while pressure at the shock front is calculated from hydrocode simulations [after convergence with measured *U*_*S*,*Qtz*_(*t*)]. During the x-ray probe period, the measured shock-front *T_MgO_*(*t*) is plotted against the calculated shock-front *P_MgO_*(*t*) in Fig. 7B. The values for all shots are listed in table S1B and are plotted in Fig. 1A and fig. S1 along with other experimental and theoretical studies.

Any tilt in the drive due to, for example, a nonuniform thickness in the glue layer between the polyimide and MgO, will cause the shock in one part of the MgO to be delayed relative to the other part of the MgO (e.g., Fig. 7A). In steady shock experiments, this does not necessarily result in an additional temperature distribution during the x-ray probe time. Nevertheless, in our analysis approach, the distribution of temperature over the SOP field of view is explicitly considered in the temperature determination and the reported uncertainty in the temperature.

#### Uncertainty in temperature

Uncertainties in the determination of temperature include the distribution of temperature states during the x-ray probe period (δ*T*_Distribution_, SD error bars in Fig. 7B). We assume that the reflectivity measured at the VISAR 532-nm probe wavelength is representative of the reflectivity over the SOP spectral range of 590 to 850 nm. We also assume that there are no reflections from the MgO/epoxy/quartz interface. On the basis of the reported shot-to-shot repeatability from previous studies (*8*, *56*), we estimate an additional random measurement uncertainty of ±300 K for all shots. The temperature uncertainties shown in Fig. 1A and fig. S1 and listed in table S1B represent the combined uncertainties (δ*T*_Total_) due to the temperature distribution during the x-ray probe period and additional random measurement uncertainties associated with the temperature measurement. These temperature uncertainties are combined as follows,

$${\delta T}_{\text{Total}}\left(K\right)=\sqrt{{{\delta T}_{\text{Distribution}}}^{2}+{{\delta T}_{\text{Random}}}^{2}}$$ (6)

Both δ*T*_Total_ and δ*T*_Distribution_ are listed in table S1B for each shot. Additional uncertainties include uncertainties in the determination of *R*(t) and systematic uncertainties related to the use of quartz as a temperature calibrant, i.e., the relationship between *U*_*S*,*Qtz*_, *R_Qtz_* and *T_Qtz_*. Systematic uncertainties are difficult to quantify, and we do not attempt to quantify individual contributions to the systematic uncertainties related to the relationships between U_*S*,*Qtz*_, *R_Qtz_*, and *T_Qtz_*, as described in Eqs. 4 and 5. However, we state the model we used, such that our temperature estimates can be reevaluated at a later time if a more accurate quartz EOS is available. Additional discussion on SOP temperature uncertainties are found in (*23*, *76*).

### Density determination

Diffraction peaks were fit with Gaussian curves to determine peak positions in 2θ and converted to atomic *d*-spacing, *d* = λ/(2sinθ), where λ is the wavelength of the He_α_ probe. MgO density, ρ*_MgO_*, is calculated individually from the B1 (002), B2 (001), and B2 (011) *d*-spacing values (fig. S2). ρ*_MgO_* for each shot is listed in table S1A. We note that there is an unexpected low rate of compression as a function of increasing pressure for the B1 and B2 phases (see also fig. S2). In our analysis, we assume the central energy of the x-ray source when calculating *d*-spacing: 8.368 keV (1.4816 Å). The x-ray source has an ∼1% spectral bandwidth (*44*). If the compressed MgO is a single crystal, as the *d*-spacing decreases, the Laue diffraction conditions to produce diffraction will only be satisfied by higher photon energies. This effect would potentially result in a slight modification of the inferred *d*-spacing values and could also be a contributing factor in the measured reduction of B1 (200) diffraction signal level as a function of pressure (Fig. 1B).

#### Uncertainty in d-spacing

Our measurement of *d*-spacing and density is based on a centroid fit to the measured diffraction peaks. As described in (*21*) a diffraction ring’s 2θ centroid can be determined in many cases to a precision of 0.05°, dependent on the ring’s location, brightness, and texture. Therefore, a single line detected at, for example, 2θ = 50° gives δd/d ≈ 9.3 × 10^−4^ Å, where δd represents the uncertainty in measured *d*-spacing. In our experiments, the reported uncertainty in measured *d*-spacing in table S1 also includes uncertainty due to: (i) accuracy of pinhole reference peaks fit to ideal ambient-pressure 2θ values, (ii) variation in *d*-spacing as a function of azimuthal angle (ϕ), and (iii) uncertainty in the sample-pinhole (reference plane) distance (*46*).

#### Uncertainty in density

Uncertainty in density (δρ) for an individual reflection from a cubic crystalline material is related to uncertainty in *d*-spacing by δρ = 3ρ(δd/d). δρ values for the (002)_*B*1_, (001)_*B*2_, and (011)_*B*2_ reflections for all shots are listed in table S1A. Assuming a normal distribution, the uncertainty in the average B2 density may be determined by

$${\delta \rho }_{B2,\mathrm{Avg}}=\sqrt{{{\delta \rho }_{B2,011}}^{2}+{{\delta \rho }_{B2,011}}^{2}+\text{Stdev}{\left({\delta \rho }_{B2,001},{\delta \rho }_{B2,011}\right)}^{2}}$$ (7)

δρ_*B*2,*Avg*_ for all shots is listed in table S1A.

#### Off-Hugoniot states generated by shock unsteadiness

In our experiments, shock unsteadiness in MgO will result in the generation of off-Hugoniot states due to either: (i) isentropic pressure-release from an initial shock state in the case of an unsupported shock or (ii) shock + ramp–compressed states in the case of a growing shock. For (i) the *P*-ρ states produced would be less dense than the Hugoniot and for (ii) the *P*-ρ states produced would be d than the Hugoniot. We note that the measured *U*_*S*,*Qtz*_(t) values in our experiments do slightly increase over time (by ∼1.5 to 3%; see fig. S3B). This may be sufficient to cause a slightly higher compressed state as compared to the shock Hugoniot.

## Supplementary Materials

### This PDF file includes:

Sections S1 and S2 Figs. S1 to S11 Table S1 References
