# Supplementary material for: B1-B2 transition in shock-compressed MgO
Source: Sci Adv. 2024 Jun 7;10(23):eadk0306. doi: 10.1126/sciadv.adk0306 (PMC11160462; doi:10.1126/sciadv.adk0306)
Supplement: Supplementary file 1 — Sections S1 and S2 Figs. S1 to S11 Table S1 References [file sciadv.adk0306_sm.pdf]

Science Advances
AAAS

# Supplementary Materials for

## B1-B2 transition in shock-compressed MgO

June K. Wicks *et al.*

Corresponding author: June K. Wicks, wicks@jhu.edu

*Sci. Adv.* **10**, eadk0306 (2024)
DOI: 10.1126/sciadv.adk0306

**This PDF file includes:**

Sections S1 and S2
Figs. S1 to S11
Table S1
References

**Supplementary Material Figures:**

**Figure S1:** P-T phase diagram of MgO.
**Figure S2:** Determined pressure-density states of shock compressed MgO [100].
**Figure S3:** Laser pulse shapes and measured quartz shock velocity profiles for all experiments.
**Figure S4:** X-ray diffraction data for all shots.
**Figure S6:** EOS models used in hydrocode calculations.
**Figure S7:** Calculated pole figures for high symmetry planes of the B2 phase as a result of the WTM mechanism.
**Figure S8:** Calculated pole figures for high symmetry planes of the B2 phase as a result of the Buerger's mechanism.
**Figure S9:** The Buerger's mechanism for the B1→B2 transformation.
**Figure S10:** Calculated strain needed for the for transformation from B1→B2.
**Figure S11:** Pressure correction of McWilliams *et al.* shock decay data.

**Supplementary Material Tables:**

**Table S1:** Summary of experimental results.

## S1. Buerger's mechanism

The Buerger's mechanism [25] is described by a uniform compression of the B1 rhombohedral primitive cell (Fig. S9) in the $[111]_{B1}$ direction and uniform expansion in all directions perpendicular to this. However, this mechanism predicts intensity distribution inconsistent with our measurements. Therefore, it was concluded that this mechanism is not activate. The stretch tensor which map the primitive (rhombohedral) B1 basis vectors, $(\vec{\mathbf{a}}_{\mathrm{P}}, \vec{\mathbf{b}}_{\mathrm{P}}, \vec{\mathbf{c}}_{\mathrm{P}})$ to the B2 basis vectors, $(\mathbf{a}', \mathbf{b}', \mathbf{c}')$ can be determined by calculating the stretches in the principal directions. These directions are the ones along which the deformation is purely hydrostatic. A stretch of 1 preserves the length of vectors in the direction, and any value higher (lower) will expand (contract) vectors in that principal direction.

The Buerger's mechanism requires $\langle 111\rangle_{B1}$ to be one such principal direction. Since there is uniform contraction in the corresponding $\{111\}_{B1}$ plane, any two orthogonal vectors in this plane can be chosen as the other two principal directions. For the example illustrated in Fig. S9, the three principal directions are $\frac{1}{\sqrt{3}}[111]_{B1}$, $\frac{1}{\sqrt{2}}[1\bar{1}0]_{B1}$ and $\frac{1}{\sqrt{6}}[11\bar{2}]_{B1}$. The amount of stretch can be calculated using the XRD measured lattice parameters as shown in Fig. S10A. The direction ($S$) and stretches ($\Lambda$) for shot s22257 (Fig. 2A, 442(28) GPa) are given by:

$$S = \begin{pmatrix} 1/\sqrt{3} & 1/\sqrt{2} & 1/\sqrt{6} \\ 1/\sqrt{3} & -1/\sqrt{2} & 1/\sqrt{6} \\ 1/\sqrt{3} & 0 & -2/\sqrt{6} \end{pmatrix}; \qquad (8)$$

$$\Lambda = \begin{pmatrix} 0.6233 & 0 & 0 \\ 0 & 1.2466 & 0 \\ 0 & 0 & 1.2466 \end{pmatrix}.$$

The stretches for the MgO B1→B2 is slightly different than the values reported for NaCl in Ref. [50] (0.6 for the $[111]_{B1}$ and 1.19 for the other two). The stretch tensor is given by $S\Lambda S^{-1}$. This tensor when applied to the rhombohedral B1 basis ($\vec{\mathbf{a}}_{\mathrm{P}} = \frac{\mathrm{a}}{2}[110]_{B1}$, $\vec{\mathbf{b}}_{\mathrm{P}} = \frac{\mathrm{a}}{2}[101]_{B1}$) and $\vec{\mathbf{c}}_{\mathrm{P}} = \frac{\mathrm{a}}{2}[011]_{B1}$ (a is the lattice parameter of the B1 phase), results in the cubic B2 basis vectors ($\mathbf{a}'$, $\mathbf{b}'$, $\mathbf{c}'$ in Fig. S9). These basis vectors in the B1 crystallographic frame are given by $\frac{\mathrm{a}'}{\sqrt{6}}[22\bar{1}]_{B1}$, $\frac{\mathrm{a}'}{\sqrt{6}}[2\bar{1}2]_{B1}$ and $\frac{\mathrm{a}'}{\sqrt{6}}[\bar{1}22]_{B1}$ ($a'$ is the lattice parameter of the B2 phase). There are a total of twelve orientation variants for this transformation mechanism. One such variant ($[\bar{1}11]_{B1}$) is schematically shown in Fig. S9.

## S2. Watanabe-Tokonami-Morimoto mechanism

The WTM mechanism, as described in the main text and Fig. 2B, produces a XRD texture pattern consistent with our data. The principal stretches, using the same method described above, depend on the XRD-determined lattice parameters for the B1 and B2 phases. In the WTM mechanism, there is expansion in the $\langle 110 \rangle_{B1}$ direction and uniform contraction in the corresponding $\{110\}_{B1}$ plane. The principal direction, $S$ (as columns) and principal stretch, $\Lambda$ (eigenvalue) along these directions for shot s22257 (Fig. 2A, 442(28) GPa) are given by,

$$S = \begin{pmatrix} -1/\sqrt{2} & 0 & 1/\sqrt{2} \\ 1/\sqrt{2} & 0 & 1/\sqrt{2} \\ 0 & 1 & 0 \end{pmatrix}; \tag{9}$$

$$\Lambda = \begin{pmatrix} 0.8785 & 0 & 0 \\ 0 & 0.8785 & 0 \\ 0 & 0 & 1.2424 \end{pmatrix}. \tag{10}$$

The stretch tensor, $F$ is given by $S\Lambda S^{-1}$. Strain values for all the other shots are presented in Fig. S10B. The WTM mechanism produces six distinct orientation variants.

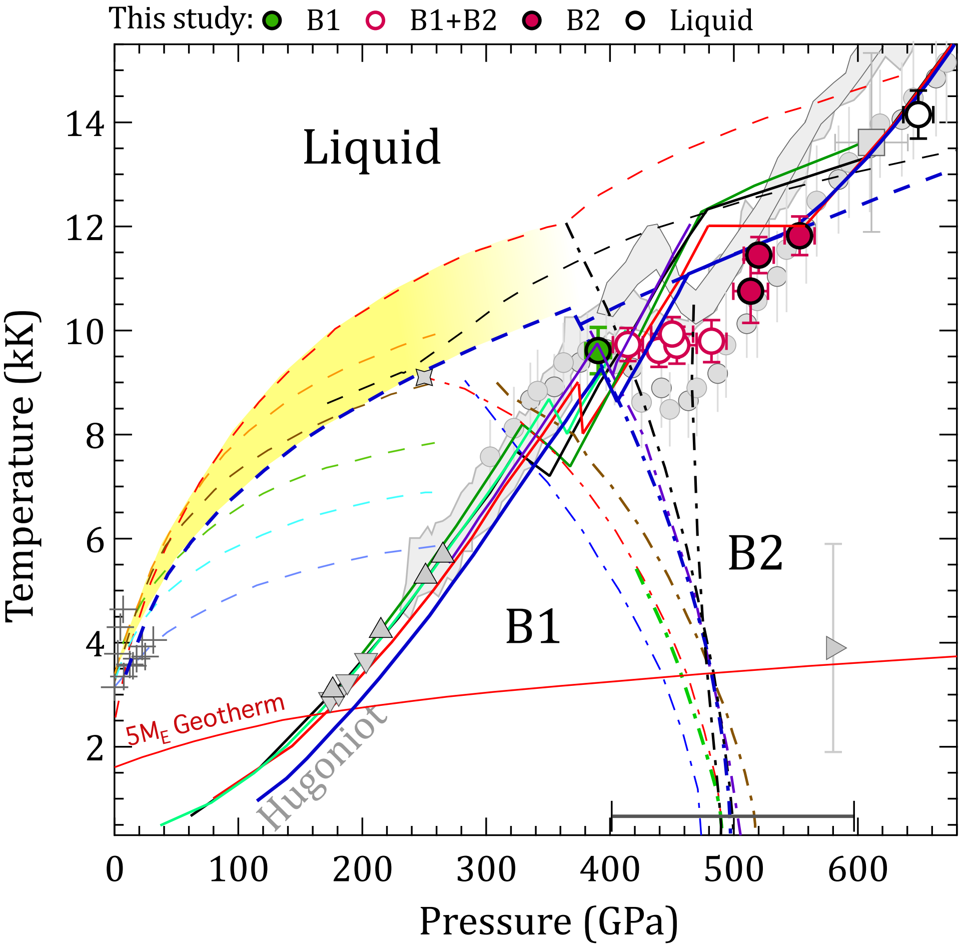


**Figure S1.** ***P-T*** **phase diagram of MgO.** Plotted are the predicted B1→B2 phase boundary (dashed-dot curves) [10, 14–18, 36, 59, 77, 78, 80, 81] and liquid phase boundaries (dashed curves) [10, 15, 17, 82–86] with shaded yellow region constrained at low-pressure by static-compression (gray crosses) [84, 87] and at 250 GPa and 9100 K by gas-gun experiments (gray diamond) [88]. We note there is an additional experimental constraint on the melt curve between 1200-2000 GPa [20]. Also plotted is the estimated onset condition for the B2-phase from laser ramp-compression techniques (gray right-triangle) [9], and an example of the modeled temperature profile within a five-Earth-mass rocky exoplanet (red curve) [89]. Predicted Hugoniots are shown as the solid traces [10, 14–16, 59, 72] and compared to previous *P-T* measurements along the Hugoniot: (gray circles [8] - which have been corrected in pressure based on the subsequent Hugoniot measurements by Root *et al.* [10], see Fig. S11), (gray bands) [19], (gray square) [59], (gray down triangle) [90], (gray up triangle) [91]. The calculated 0-K range of onset pressures for the B1→B2 phase transformation is shown by the scale on the bottom axis (~400-600 GPa) [92]. The data reported in our combined laser-shock compression, X-ray diffraction and pyrometry study are shown as circles (see also Fig. 1a).

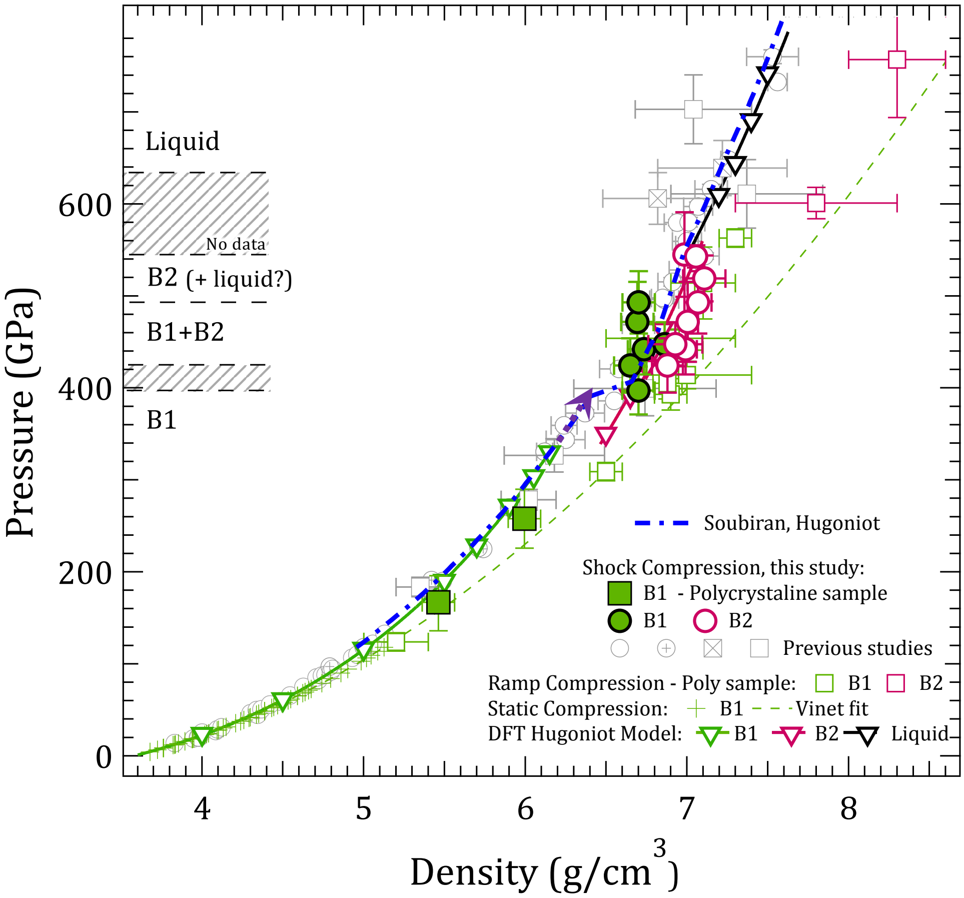


**Figure S2. Determined pressure-density states of shock compressed MgO [100].** A summary of published P-$\rho$ data for MgO. Calculated pressure and measured density for the B1 (green filled circles) and B2-phase (maroon open circles) (see Materials and Methods). Hugoniot data based on shock-speed measurements are shown as the open gray circle, crossed circle, open square and crossed square symbols [2, 8, 10, 58–61]. A Hugoniot based on density functional theory (DFT) calculations is shown as the green (B1), maroon (B2) and black (liquid) open triangles [10]. Solid line fits to these points are based on linear fits in $U_s$-$u_p$. The Hugoniot calculated from quasi-harmonic *ab initio* molecular dynamic calculations from Soubiran *et al.* [16] are shown as the blue dash-dot curve). The purple dashed arrow represents an extension of the B1 phase up to pressure where we see only B1 in our X-ray diffraction (XRD) data. Ramp compression XRD data is shown as open green (B1) and maroon (B2) squares [9], and static compression (B1) data is shown as green crosses [30] with a Vinet equation-of-state (EOS) fit to high pressure (green dashed). In our experiments, the pressure regions associated with XRD-measured phases are shown by the left axis. The shaded regions represent pressure intervals where no data was obtained. At 425 GPa, measured densities show divergence from previous Hugoniot measurements with increased agreement at higher pressures (~600 GPa) (see also Fig. 3B).

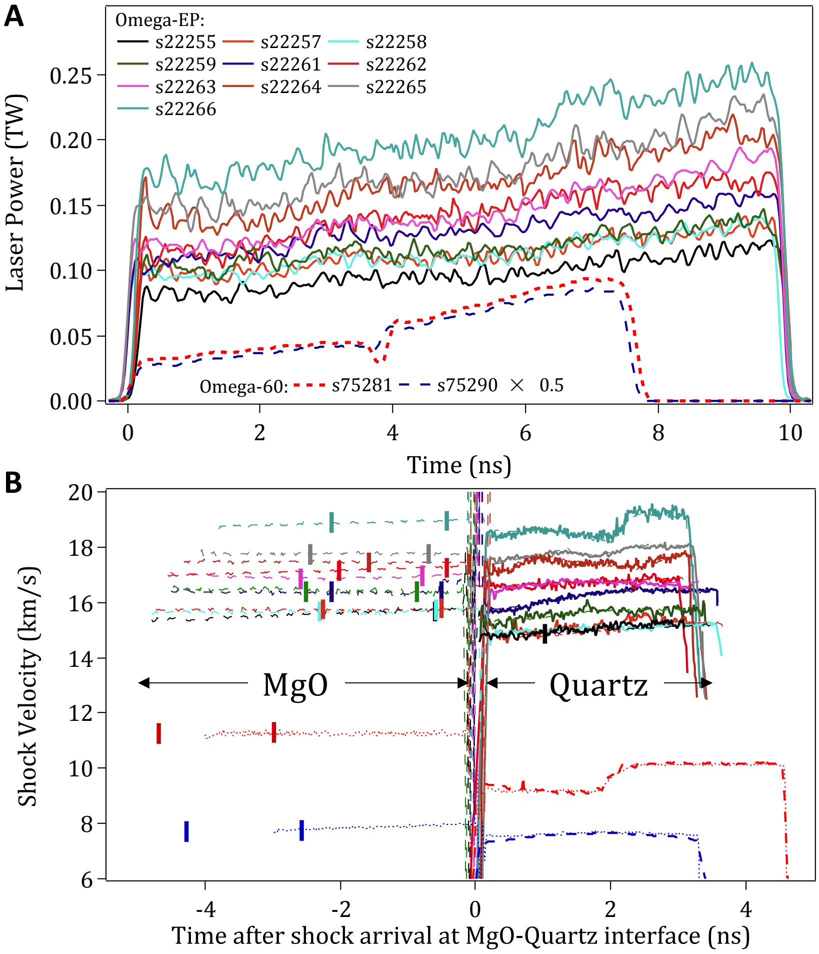


**Figure S3. Laser pulse shapes and measured quartz shock velocity profiles for all experiments.** **(A)** Laser pulse shapes and **(B)** the associated measured quartz shock velocity, $U_{S,Qtz}$, for all the shots considered in our study (bold solid and bold dashed curves). The light dashed and light dotted curves represent HYADES hydrocode simulations fit to the measured $U_{S,Qtz}$, which provides information on the $U_{S,MgO}$ states during the x-ray probe period (shown here as bound by vertical bars).

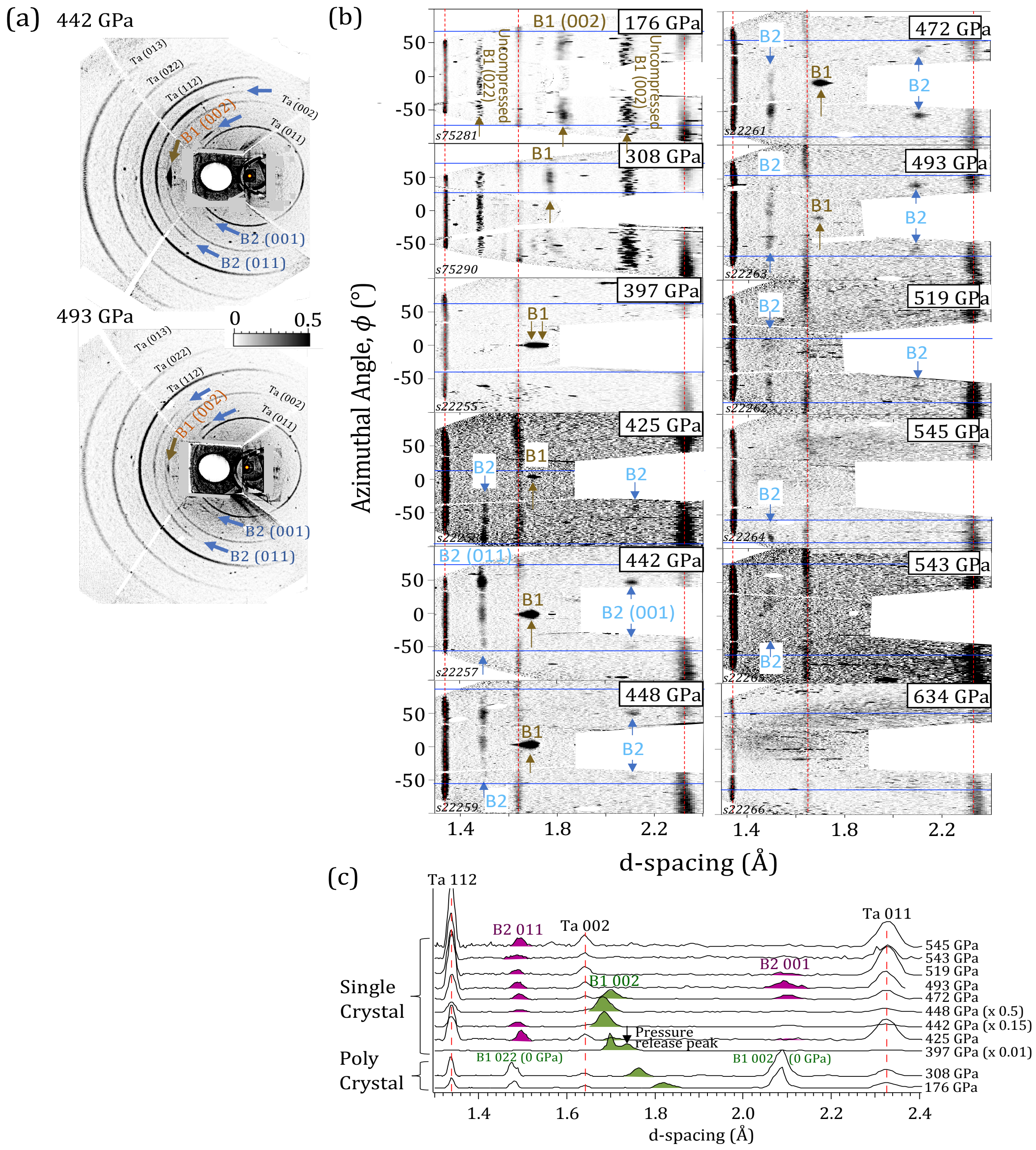


**Figure S4. X-ray diffraction data for all shots. (A)** Stereographic projection of X-ray diffraction data for two representative shots, demonstrating a repeatable texture, and the drop in diffraction intensity, as a function of increasing pressure. Peak photo-stimulated luminescence (PSL) counts for the B1 (002) peak is 145 (top at 442(28) GPa) and 0.8 (bottom at 493(34) GPa). **(B)** Image plates detectors projected as a function of $\phi$, and $d$-spacing for all shots within this study [45]. The red vertical dashed lines represent the positions of the reference peaks from the ambient-pressure Ta pinhole. The textured peaks for the MgO B1- and B2-phase are indicated. For the two lowest pressure shots (176(31) and 308(32) GPa) polycrystalline MgO samples were used. For all other shots single crystal MgO [100] samples were used. **(C)** Lineouts integrated between horizontal blue lines on panels in (b) show Ta reference peaks, B1 and B2 peaks.

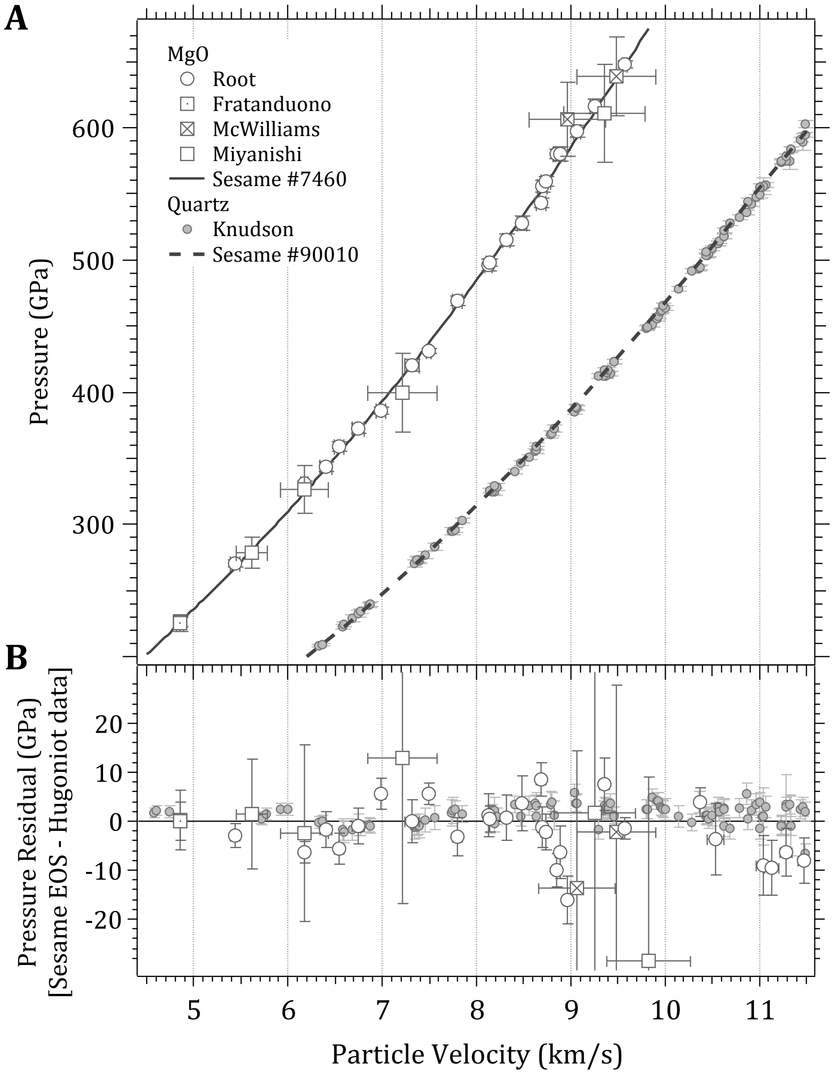


**Figure S5. EOS models used in hydrocode calculations.** **(A)** Summary of tabulated Hugoniot models used in the hydrocode determination of MgO pressure [64] versus measured Hugoniot density for MgO [2, 8, 10, 58–61] and quartz [65]. **(B)** Pressure residuals of Sesame EOS model Hugoniots – Hugoniot data.

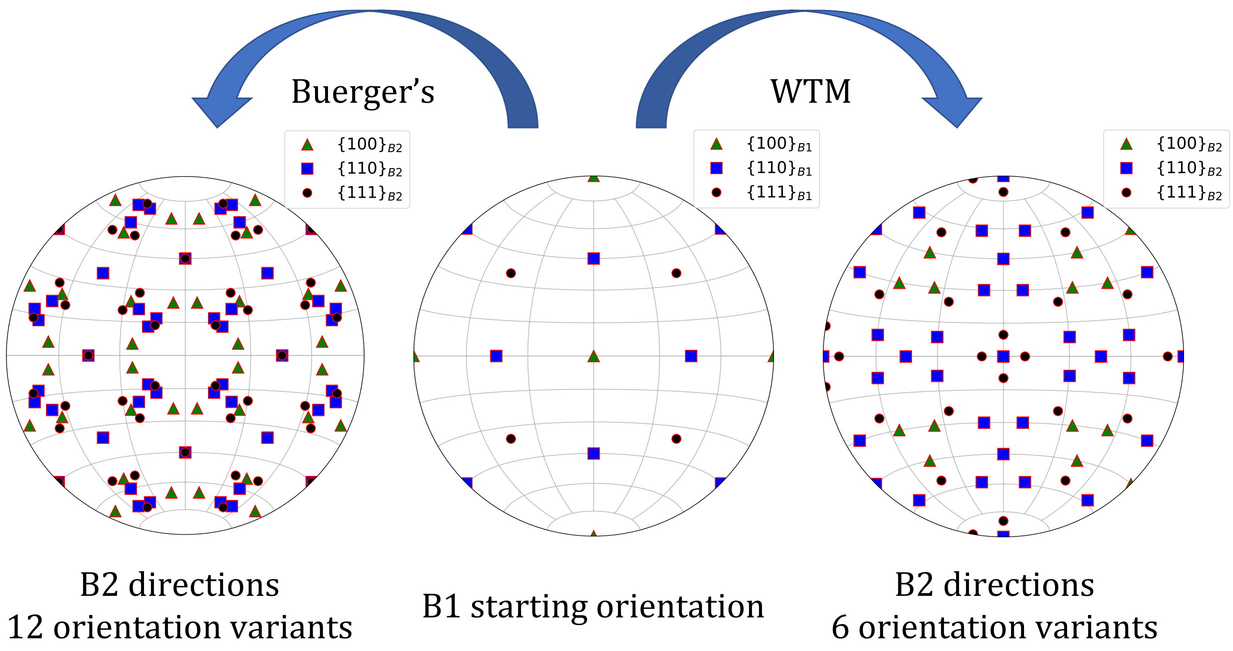


**Figure S6. Pole figure**.

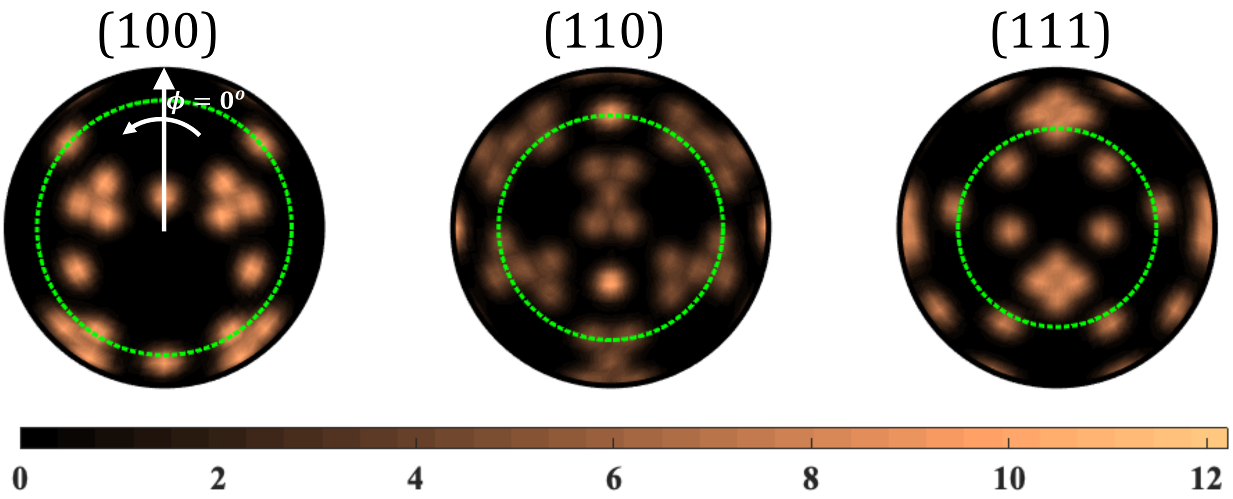


**Figure S7. Calculated pole figures for high symmetry planes of the B2 phase as a result of the WTM mechanism.** The observable $2\theta$ ring in our experimental geometry is shown by the green circle.

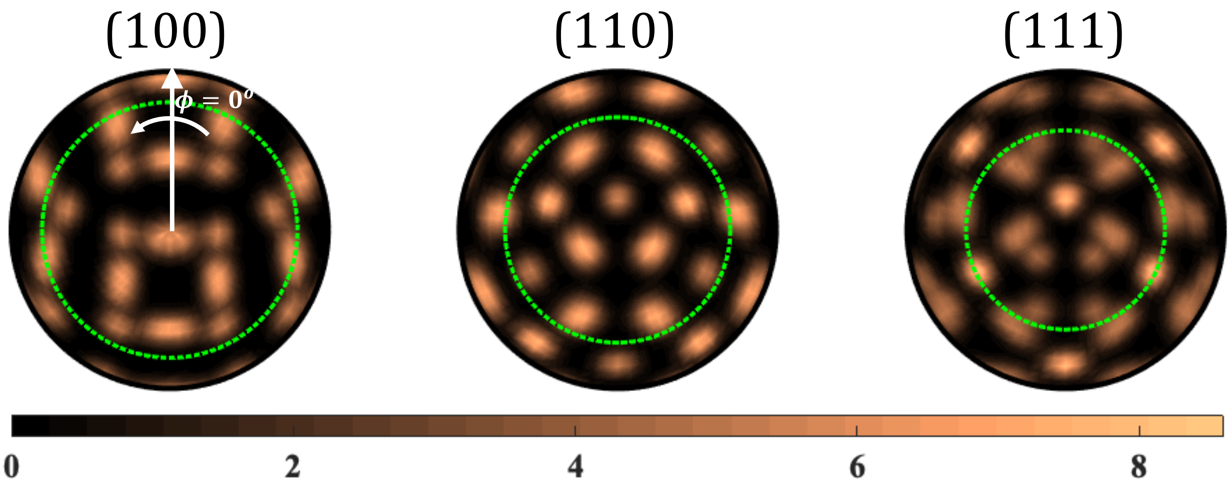


**Figure S8. Calculated pole figures for high symmetry planes of the B2 phase as a result of the Buerger's mechanism.** The observable $2\theta$ ring in our experimental geometry is shown by the green circle.

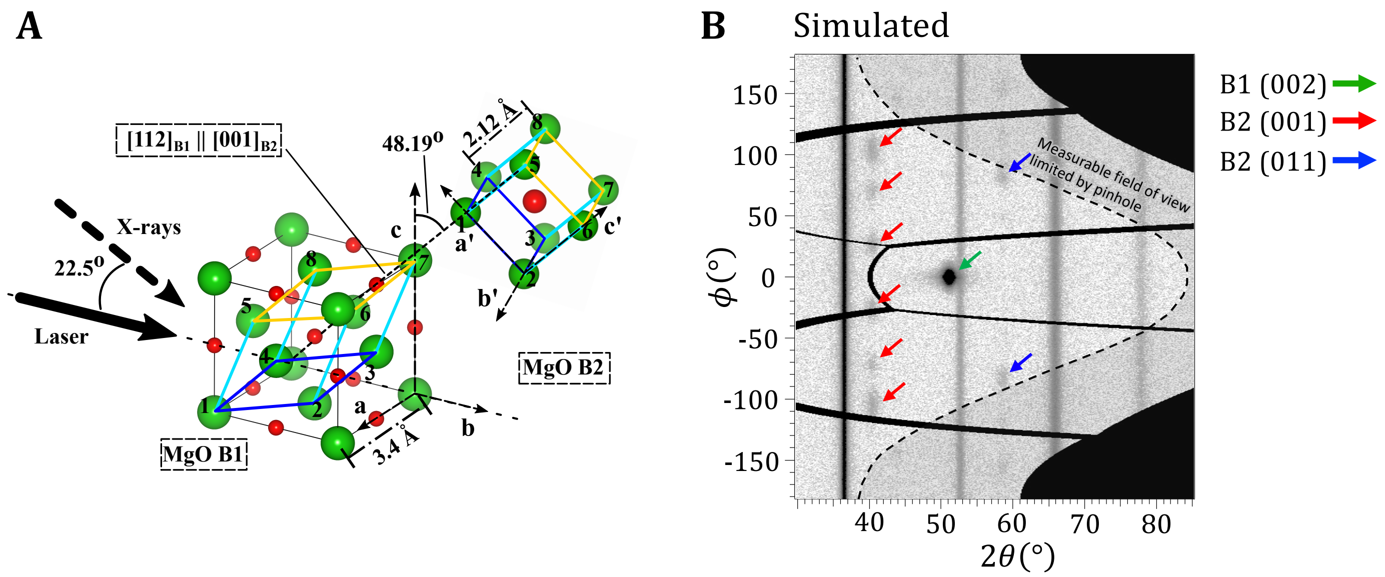


**Figure S9. The Buerger's mechanism for the B1→B2 transformation. A.** The Buerger's mechanism for the B1→B2 transformation describes compression and rotation of the B1 rhombohedral primitive cell [25] with an orientation relationship of $[112]_{B1}||[001]_{B2}$ and $[1\bar{1}0]_{B1}||[110]_{B2}$. **B.** Using the forward model, for the same grain mosaicity used for simulation Fig. 2A, the resulting diffraction pattern has very different intensity distribution of the B2 phase. Therefore, it was concluded that the Buerger's mechanism is not active.

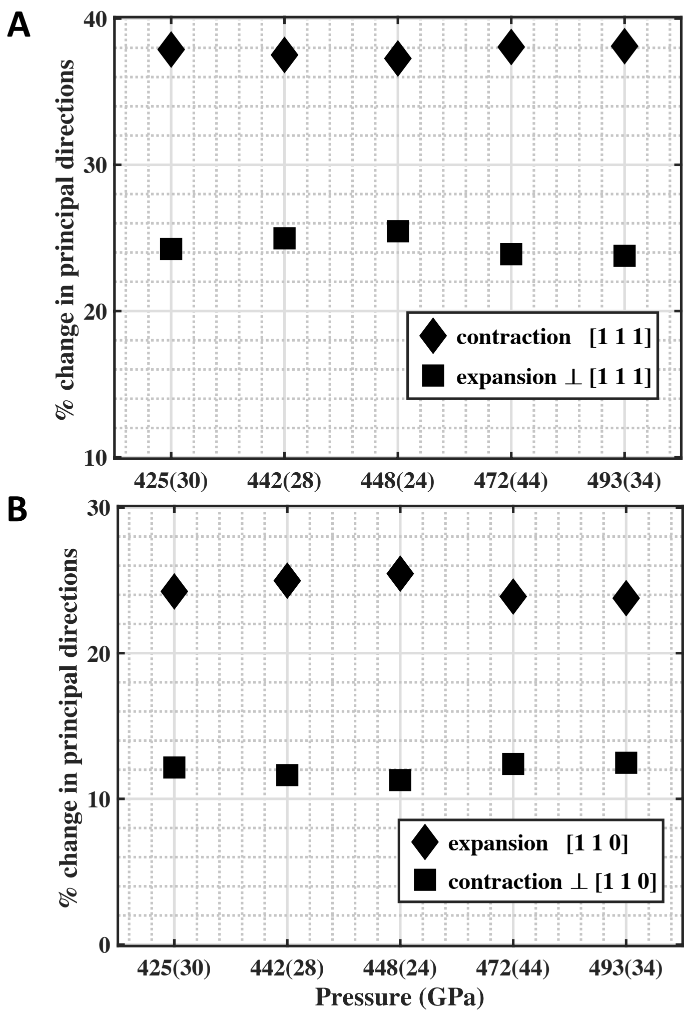


**Figure S10. Calculated strain needed for the for transformation from B1→B2**. Here, shown for the **(A)** the Buerger's mechanism [25] and **(B)** the ideal WTM mechanism [26]. The pressures for five shots are represented on the x-axis.

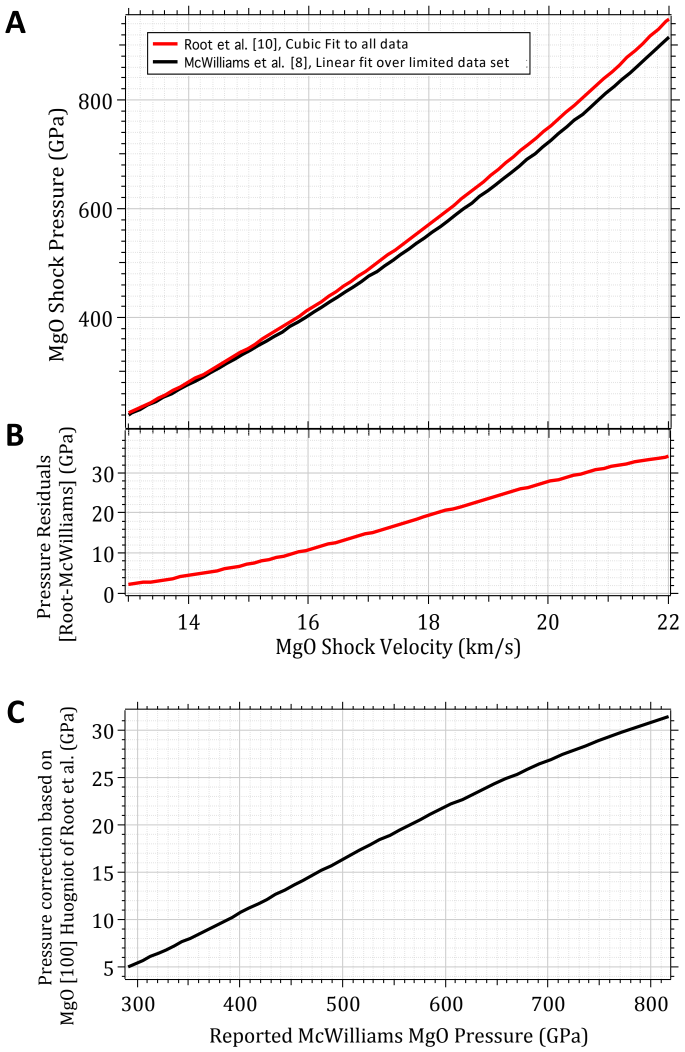


**Figure S11. Pressure correction of McWilliams *et al.* shock decay data [8] based on updated MgO [100] Hugoniot data by Root *et al.* [10]. (A) (B) (C).**

## (A) X-ray Diffraction

| P (GPa) | Shot No. | *d*-spacing[a] (Å) B1 (002) | *d*-spacing[a] (Å) B2 (001) | *d*-spacing[a] (Å) B2 (011) | Density (g/cm³) B1 (002) | Density (g/cm³) B2 (001) | Density (g/cm³) B2 (011) | Density (g/cm³) B2 Average[#] | Normalized B1 Intensity[@] |
|---|---|---|---|---|---|---|---|---|---|
| 176(31)[b] | s75281 | 1.830(3) | - | - | 5.462(36) | - | - | | |
| 308(32)[b] | s75290 | 1.774(3) | - | - | 5.995(36) | - | - | | |
| 397(26) | s22255 | 1.709(3) | - | - | 6.746(36) | - | - | | 1 |
| 425(30) | s22258 | 1.714(2) | 2.141(2) | 1.505(2) | 6.650(23) | 6.817(19) | 6.942(28) | 6.880(95) | |
| 442(28) | s22257 | 1.707(3) | 2.125(3) | 1.500(4) | 6.734(36) | 6.975(30) | 7.017(56) | 6.996(70) | 0.275 |
| 448(24) | s22259 | 1.696(7) | 2.131(14) | 1.505(7) | 6.862(85) | 6.921(136) | 6.941(97) | 6.931(168) | 0.264 |
| 472(44) | s22261 | 1.710(2) | 2.121(3) | 1.501(2) | 6.693(23) | 7.016(30) | 6.999(28) | 7.007(43) | 0.064 |
| 493(34) | s22263 | 1.710(1) | 2.111(4) | 1.499(3) | 6.702(12) | 7.106(40) | 7.021(42) | 7.063(84) | 0.002 |
| 519(25) | s22262 | - | 2.109(5) | 1.495(8) | - | 7.136(51) | 7.080(114) | 7.108(131) | |
| 543(12) | s22265 | - | - | 1.497(2) | - | - | 7.057(28) | 7.057(28) | |
| 545(46) | s22264 | - | - | 1.502 (1) | - | - | 6.985(14) | 6.985(14) | |
| 634(29) | s22266 | - | - | - | - | - | - | | |

## (B) Pyrometry

| P (GPa) | Shot No. | T($\delta T_{Total}$) (K) | $\delta T_{Distribution}$ (K) |
|---|---|---|---|
| - | s75281 | - | |
| - | s75290 | - | |
| 390(10) | s22255 | 9614(450) | 330 |
| 414(12) | s22258 | 9731(320) | 110 |
| 439(16) | s22257 | 9621(320) | 110 |
| 454(16) | s22259 | 9717(360) | 190 |
| 450(17) | s22261 | 9933(330) | 130 |
| 482(12) | s22263 | 9796(400) | 270 |
| 513(14) | s22262 | 10757(610) | 530 |
| 520(12) | s22264 | 11450(350) | 170 |
| 553(12) | s22265 | 11822(370) | 220 |
| 649(12) | s22266 | 14152(460) | 350 |

Table S1. **Summary of experimental results.** **(A)** Measured X-ray diffraction crystal structure and density versus estimated bulk pressure. [a]Uncertainty in d-spacing includes: (i) accuracy of pinhole reference peaks fit to ideal ambient-pressure $2\theta$ values, (ii) variation in d-spacing as a function of azimuthal angle ($\phi$), and (iii) uncertainty in the sample - pinhole (reference plane) distance. Pressure uncertainty includes experimental (as determined by the VISAR record) and systematic (uncertainty in material EOS models) contributions. [b]Polycrystalline samples. [#]Values plotted in Fig. 3B. [@]Values plotted in Fig. 1B. The value for shot s22258 was deemed unreliable due to target misalignment. **(B)** Measured average shock-front temperature and calculated average shock-front pressure, during the X-ray probe period. The total uncertainties in temperature ($\delta T_{Total}$) represent the standard deviation in the measured temperature distribution ($\delta T_{Distribution}$) (see Fig. 7B), combined with an additional ± 300 K estimated to represent random uncertainties in the measurement of temperature (see Fig. 1, and Methods and Materials).

## REFERENCES AND NOTES

1. M. Mehl, R. Hemley, L. Boyer, Potential-induced breathing model for the elastic moduli and high-pressure behavior of the cubic alkaline-earth oxides. *Phys. Rev. B* **33**, 8685–8696 (1986).

2. T. S. Duffy, R. J. Hemley, H.-K. Mao, Equation of state and shear strength at multimegabar pressures: Magnesium oxide to 227 GPa. *Phys. Rev. Lett.* **74**, 1371–1374 (1995).

3. T. Irifune, T. Tsuchiya, Phase transitions and mineralogy of the lower mantle, in *Treatise on Geophysics* (Elsevier BV, ed. 2, 2015).

4. F. Coppari, R. Smith, J. Wang, M. Millot, D. Kim, J. Rygg, S. Hamel, J. Eggert, T. Duffy, Implications of the iron oxide phase transition on the interiors of rocky exoplanets. *Nat. Geosci.* **14**, (2021).

5. R. J. Spaargaren, H. S. Wang, S. J. Mojzsis, M. D. Ballmer, P. J. Tackley, Plausible constraints on the range of bulk terrestrial exoplanet compositions in the solar neighborhood. *Astrophys. J.* **948**, 53 (2023).

6. J. Amodeo, S. Merkel, C. Tromas, P. Carrez, S. Korte-Kerzel, P. Cordier, J. Chevalier, Dislocations and plastic deformation in MgO crystals: A review. *Crystals* **8**, 240 (2018).

7. P. Toledano, K. Knorr, L. Ehm, W. Depmeier, Phenomenological theory of the reconstructive phase transition between the NaCl and CsCl structure types. *Phys. Rev. B* **67**, 144106 (2003).

8. R. S. McWilliams, D. K. Spaulding, J. H. Eggert, P. M. Celliers, D. G. Hicks, R. F. Smith, G. W. Collins, R. Jeanloz, Phase transformations and metallization of magnesium oxide at high pressure and temperature. *Science* **338**, 1330–1333 (2012).

9. F. Coppari, R. F. Smith, J. H. Eggert, J. Wang, J. R. Rygg, A. Lazicki, J. A. Hawreliak, G. W. Collins, T. S. Duffy, Experimental evidence for a phase transition in magnesium oxide at exoplanet pressures. *Nat. Geosci.* **6**, 926–929 (2013).

10. S. Root, L. Shulenburger, R. W. Lemke, D. H. Dolan, T. R. Mattsson, M. P. Desjarlais, Shock response and phase transitions of MgO at planetary impact conditions. *Phys. Rev. Lett.* **115**, 198501 (2015).

11. F. Wagner, N. Tosi, F. Sohl, H. Rauer, T. Spohn, Rocky super-Earth interiors. *Astron. Astrophys.* **541**, A103 (2012).

12. S.-I. Karato, Rheological structure of the mantle of a super-Earth: Some insights from mineral physics. *Icarus* **212**, 14–23 (2011).

13. S. Ritterbex, T. Harada, T. Tsuchiya, Vacancies in MgO at ultrahigh pressure: About mantle rheology of super-Earths. *Icarus* **305**, 350–357 (2018).

14. J. Bouchet, F. Bottin, V. Recoules, F. Remus, G. Morard, R. Bolis, A. Benuzzi-Mounaix, Ab initiocalculations of the B1-B2 phase transition in MgO. *Phys. Rev. B* **99**, 094113 (2019).

15. F. Soubiran, B. Militzer, Electrical conductivity and magnetic dynamos in magma oceans of Super-Earths. *Nat. Commun.* **9**, 3883 (2018).

16. F. Soubiran, B. Militzer, Anharmonicity and phase diagram of magnesium oxide in the megabar regime. *Phys. Rev. Lett.* **125**, 175701 (2020).

17. B. Boates, S. A. Bonev, Demixing instability in dense molten MgSiO3 and the phase diagram of MgO. *Phys. Rev. Lett.* **110**, 135504 (2013).

18. A. R. Oganov, P. I. Dorogokupets, All-electron and pseudopotential study of MgO: Equation of state, anharmonicity, and stability. *Phys. Rev. B* **67**, 224110 (2003).

19. R. Bolis, G. Morard, T. Vinci, A. Ravasio, E. Bambrink, M. Guarguaglini, M. Koenig, R. Musella, F. Remus, J. Bouchet, N. Ozaki, K. Miyanishi, T. Sekine, Y. Sakawa, T. Sano, R. Kodama, F. Guyot, A. Benuzzi-Mounaix, Decaying shock studies of phase transitions in MgO-SiO2systems: Implications for the super-Earths' interiors. *Geophys. Res. Lett.* **43**, 9475–9483 (2016).

20. L. Hansen, D. Fratanduono, S. Zhang, D. Hicks, T. Suer, Z. Sprowal, M. Huff, X. Gong, B. Henderson, D. Polsin, M. Zaghoo, S. X. Hu, G. W. Collins, J. R. Rygg, Melting of magnesium oxide up to two terapascals using double-shock compression. *Phys. Rev. B.* **104**, 014106 (2021).

21. J. R. Rygg, J. H. Eggert, A. E. Lazicki, F. Coppari, J. A. Hawreliak, D. G. Hicks, R. F. Smith, C. M. Sorce, T. M. Uphaus, B. Yaakobi, G. W. Collins, Powder diffraction from solids in the terapascal regime. *Rev. Sci. Instrum.* **83**, 113904 (2012).

22. P. M. Celliers, M. Millot, Imaging velocity interferometer system for any reflector (VISAR) diagnostics for high energy density sciences. *Rev. Sci. Instrum.* **94**, 011101 (2023).

23. M. Gregor, R. Boni, A. Sorce, J. Kendrick, C. McCoy, D. Polsin, T. Boehly, P. Celliers, G. Collins, D. Fratanduono, J. Eggert, M. Millot, Absolute calibration of the OMEGA streaked optical pyrometer for temperature measurements of compressed materials. *Rev. Sci. Instrum.* **87**, 114903 (2016).

24. H. T. Stokes, D. M. Hatch, Procedure for obtaining microscopic mechanisms of reconstructive phase transitions in crystalline solids. *Phys. Rev. B* **65**, 144114 (2002). https://doi.org/10.1103/PhysRevB.65.144114.

25. M. Buerger, *Phase Transformations in Solids* (Wiley, New York, 1951), chap. 6.

26. M. Watanabe, M. Tokonami, N. Morimoto, The transition mechanism between the CsCl-type and NaCl-type structures in CsCl. *Acta Crystallogr. A* **33**, 294–298 (1977).

27. B. Hyde, M. O'Keeffe, *Phase Transitions*, L. E. Cross, Ed. (Pergamon Press, Oxford, 1973).

28. Y. Y. Zhang, Y. X. Li, D. Fan, N. B. Zhang, J. W. Huang, M. X. Tang, Y. Cai, X. L. Zeng, T. Sun, K. Fezzaa, S. Chen, S. N. Luo, Ultrafast x-ray diffraction visualization of *B*1–*B*2 phase transition in KCl under shock compression. *Phys. Rev. Lett.* **127**, 045702 (2021).

29. M. Catti, Ab initiopredicted metastable TlI-like phase in theB1toB2high-pressure transition of CaO. *Phys. Rev. B* **68**, 100101 (2003).

30. S. D. Jacobsen, C. M. Holl, K. A. Adams, R. A. Fischer, E. S. Martin, C. R. Bina, J.-F. Lin, V. B. Prakapenka, A. Kubo, P. Dera, Compression of single-crystal magnesium oxide to 118 GPa and a ruby pressure gauge for helium pressure media. *Am. Mineral.* **93**, 1823–1828 (2008).

31. B. J. Jensen, G. Gray III, R. S. Hixson, Direct measurements of the α-ϵ transition stress and kinetics for shocked iron. *J. Appl. Phys.* **105**, 103502 (2009).

32. R. F. Smith, J. H. Eggert, D. C. Swift, J. Wang, T. S. Duffy, D. G. Braun, R. E. Rudd, D. B. Reisman, J.-P. Davis, M. D. Knudson, G. W. Collins, Time-dependence of the alpha to epsilon phase transformation in iron. *J. Appl. Phys.* **114**, 223507 (2013).

33. J. Christian, *The Theory of Transformations in Metals and Alloys*, (Pergamon, Oxford, 1965).

34. D. Zahn, S. Leoni, Nucleation and growth in pressure-induced phase transitions from molecular dynamics simulations: Mechanism of the reconstructive transformation of NaCl to the CsCl-type structure. *Phys. Rev. Lett.* **92**, 250201 (2004).

35. A. R. Oganov, *Modern Methods of Crystal Structure Prediction* (John Wiley & Sons, 2010).

36. S. Zhang, R. Paul, S. Hu, M. A. Morales, Toward an accurate equation of state and B1-B2 phase boundary for magnesium oxide up to terapascal pressures and electron-volt temperatures. *Physical Review B* **107**, 224109 (2023).

37. C. Kwon, Y. Xia, F. Zhou, B. Han, Dominant effect of anharmonicity on the equation of state and thermal conductivity of MgO under extreme conditions. *Phys. Rev. B* **102**, 184309 (2020).

38. G. Ackland, Elastic properties as a pointer to phase transitions. *RIKEN Rev.*, 34–38 (2000).

39. S. Singh, R. Briggs, M. G. Gorman, L. X. Benedict, C. J. Wu, S. Hamel, A. L. Coleman, F. Coppari, A. Fernandez-Panella, C. McGuire, M. Sims, J. K. Wicks, J. H. Eggert, D. E. Fratanduono, R. F. Smith, Structural study of hcp and liquid iron under shock compression up to 275 GPa. *Phys. Rev. B* **108**, 184104 (2023). https://link.aps.org/doi/10.1103/PhysRevB.108.184104.

40. S. Singh, M. G. Gorman, P. G. Heighway, J. V. Bernier, D. McGonegle, H. J. Lee, B. Nagler, J. H. Eggert, R. F. Smith, Measurement of competing pathways in a shock-induced phase transition in zirconium by femtosecond diffraction, arXiv:2310.07035 [cond-mat.mtrl-sci] (2023).

41. V. I. Levitas, R. Ravelo, Virtual melting as a new mechanism of stress relaxation under high strain rate loading. *Proc. Natl. Acad. Sci.* **109**, 13204–13207 (2012).

42. M. M. Budzevich, V. V. Zhakhovsky, C. T. White, I. I. Oleynik, Evolution of shock-induced orientation-dependent metastable states in crystalline aluminum. *Phys. Rev. Lett.* **109**, 125505 (2012).

43. T. S. Duffy, T. J. Ahrens, Compressional sound velocity, equation of state, and constitutive response of shock-compressed magnesium oxide. *J. Geophys. Res.* **100**, 529–542 (1995).

44. F. Coppari, R. Smith, D. Thorn, J. Rygg, D. Liedahl, R. Kraus, A. Lazicki, M. Millot, J. Eggert, Optimized x-ray sources for x-ray diffraction measurements at the omega laser facility. *Rev. Sci. Instrum.* **90**, 125113 (2019).

45. J. K. Wicks, R. F. Smith, D. E. Fratanduono, F. Coppari, R. G. Kraus, M. G. Newman, J. R. Rygg, J. H. Eggert, T. S. Duffy, Crystal structure and equation of state of Fe-Si alloys at super-Earth core conditions. *Sci. Adv.* **4**, eaao5864 (2018).

46. J. R. Rygg, R. F. Smith, A. E. Lazicki, D. G. Braun, D. E. Fratanduono, R. G. Kraus, J. M. McNaney, D. C. Swift, C. E. Wehrenberg, F. Coppari, M. F. Ahmed, M. A. Barrios, K. J. M. Blobaum, G. W. Collins, A. L. Cook, P. Di Nicola, E. G. Dzenitis, S. Gonzales, B. F. Heidl, M. Hohenberger, A. House, N. Izumi, D. H. Kalantar, S. F. Khan, T. R. Kohut, C. Kumar, N. D. Masters, D. N. Polsin, S. P. Regan, C. A. Smith, R. M. Vignes, M. A. Wall, J. Ward, J. S. Wark, T. L. Zobrist, A. Arsenlis, J. H. Eggert, X-ray diffraction at the National Ignition Facility. *Rev. Sci. Instrum.* **91**, 043902 (2020). https://doi.org/10.1063/1.5129698.

47. N. R. Barton, D. E. Boyce, P. R. Dawson, Pole figure inversion using finite elements over Rodrigues space. *Textures Microstruct* **35**, 113–144 (2002).

48. J. V. Bernier, M. P. Miller, D. E. Boyce, A novel optimization-based pole-figure inversion method: Comparison with WIMV and maximum entropy methods. *J. Appl. Cryst.* **39**, 697–713 (2006).

49. S. Singh, D. E. Boyce, J. V. Bernier, N. R. Barton, Discrete spherical harmonic functions for texture representation and analysis. *J. Appl. Cryst.* **53**, 1299–1309 (2020).

50. W. L. Fraser, S. W. Kennedy, The crystal-structural transformation NaCl-type → CsCl-type: Analysis by martensite theory. *Acta Crystallogr. A* **30**, 13–22 (1974).

51. H. D. Bellamy, E. H. Snell, J. Lovelace, M. Pokross, G. E. Borgstahl, The high-mosaicity illusion: Revealing the true physical characteristics of macromolecular crystals. *Acta Crystallogr. D Biol. Crystallogr.* **56**, 986–995 (2000).

52. K. Ichiyanagi, S. Takagi, N. Kawai, R. Fukaya, S. Nozawa, K. G. Nakamura, K.-D. Liss, M. Kimura, S.-I. Adachi, Microstructural deformation process of shock-compressed polycrystalline aluminum. *Sci. Rep.* **9**, 1–8 (2019).

53. P. M. Celliers, D. K. Bradley, G. W. Collins, D. G. Hicks, T. R. Boehly, W. J. Armstrong, Line-imaging velocimeter for shock diagnostics at the OMEGA laser facility. *Rev. Sci. Instrum.* **75**, 4916–4929 (2004).

54. A. N. Mostovych, Y. Chan, Reflective probing of the electrical conductivity of hot aluminum in the solid, liquid, and plasma phases. *Phys. Rev. Lett.* **79**, 5094–5097 (1997).

55. D. Hicks, T. Boehly, P. Celliers, J. Eggert, E. Vianello, D. Meyerhofer, G. Collins, Shock compression of quartz in the high-pressure fluid regime. *Phys. Plasmas* **12**, 082702 (2005).

56. M. Millot, N. Dubrovinskaia, A. Cernok, S. Blaha, L. Dubrovinsky, D. Braun, P. Celliers, G. Collins, J. Eggert, R. Jeanloz, Shock compression of stishovite and melting of silica at planetary interior conditions. *Science* **347**, 418–420 (2015).

57. J. T. Larsen, S. M. Lane, HYADES—A plasma hydrodynamics code for dense plasma studies. *J. Quant. Spectrosc. Ra.* **51**, 179–186 (1994).

58. S. P. Marsh, *LASL Shock Hugoniot Data* (University of California Press, 1980).

59. K. Miyanishi, Y. Tange, N. Ozaki, T. Kimura, T. Sano, Y. Sakawa, T. Tsuchiya, R. Kodama, Laser-shock compression of magnesium oxide in the warm-dense matter regime. *Phys. Rev. E* **92**, 023103 (2015).

60. D. E. Fratanduono, J. H. Eggert, M. C. Akin, R. Chau, N. C. Holmes, A novel approach to Hugoniot measurements utilizing transparent crystals. *J. Appl. Phys.* **114**, 043518 (2013).

61. M. Van Thiel, *Compendium of Shock Wave Data, Lawrence Livermore Laboratory Technical Report* (UCRL-50108, 1977), pp. 401–405.

62. G. R. Fowles, Shock wave compression of hardened and annealed 2024 aluminum. *J. Appl. Phys.* **32**, 1475–1487 (1961).

63. R. Hill, *The Mathematical Theory of Plasticity* (Oxford Univ. Press, 1998), vol. 11.

64. S. P. Lyon, J. D. Johnson, Sesame: the Los Alamos National Laboratory equation of state database, Los Alamos National Laboratory, Los Alamos, NM, LA-UR-92-3407 (1992).

65. M. Knudson, M. Desjarlais, Adiabatic release measurements inα-quartz between 300 and 1200 GPa: Characterization of α-quartz as a shock standard in the multimegabar regime. *Phys. Rev. B* **88**, 184107 (2013).

66. D. Zahn, O. Hochrein, S. Leoni, Multicenter multidomainB1−B2pressure-induced reconstructive phase transition in potassium fluoride. *Phys. Rev. B* **72**, 094106 (2005).

67. L. Barker, R. Hollenbach, Shock wave study of the α ↔ ε phase transition in iron. *J. Appl. Phys.* **45**, 4872–4887 (1974).

68. M. G. Gorman, S. J. Ali, P. M. Celliers, J. L. Peebles, D. J. Erskine, J. M. McNaney, J. H. Eggert, R. F. Smith, Measurement of shock roughness due to phase plate speckle imprinting relevant for x-ray diffraction experiments on 3rd and 4th generation light sources. *J. Appl. Phys.* **132**, 175902 (2022).

69. A. Krygier, C. Wehrenberg, J. Bernier, S. Clarke, A. Coleman, F. Coppari, T. Duffy, M. Gorman, M. Hohenberger, D. Kalantar, G. E. Kemp, A. L. Coleman, S. F. Khan, F. Coppari, C. Krauland, T. S. Duffy, R. G. Kraus, A. Lazicki, M. Millot, C. Yeamans, M. J. MacDonald, A. G. MacPhee, Y. Ping, P. L. Poole, J. R. Rygg, E. Marley, M. C. Marshall, M. Schneider, H. Sio, M. May, J. M. McNaney, S. Stoupin, D. Swift, T. Zobrist, R. F. Smith, J. H. Eggert, X-ray source characterization and sample heating on x-ray diffraction experiments at the National Ignition Facility. *Phys. Plasmas* **29**, 103302 (2022).

70. R. Qiu, Q. Zeng, K. Chen, X. Yu, J. Dai, Thermal conductivity of MgO in giant planetary interior conditions predicted by deep potential, arXiv:2310.18876 [astro-ph.EP] (2023).

71. X. Zhang, S. Li, A. Wang, H. Bao, Pressure-dependent thermal conductivity in Al, W, and Pt: Role of electrons and phonons. *Phys. Rev. B* **106**, 094313 (2022).

72. D. Cebulla, R. Redmer, Ab initiosimulations of MgO under extreme conditions. *Phys. Rev. B* **89**, 134107 (2014).

73. M. Millot, S. Hamel, J. R. Rygg, P. M. Celliers, G. W. Collins, F. Coppari, D. E. Fratanduono, R. Jeanloz, D. C. Swift, J. H. Eggert, Experimental evidence for superionic water ice using shock compression. *Nat. Phys.* **14**, 297–302 (2018).

74. S. Brygoo, M. Millot, P. Loubeyre, A. E. Lazicki, S. Hamel, T. Qi, P. M. Celliers, F. Coppari, J. H. Eggert, D. E. Fratanduono, D. G. Hick, J. R. Rygg, R. F. Smith, D. C. Swift, G. W. Collins, R. Jeanloz, Analysis of laser shock experiments on precompressed samples using a quartz reference and application to warm dense hydrogen and helium. *J. Appl. Phys.* **118**, 195901 (2015).

75. D. G. Hicks, T. R. Boehly, J. H. Eggert, J. E. Miller, P. M. Celliers, G. W. Collins, Dissociation of liquid silica at high pressures and temperatures. *Phys. Rev. Lett.* **97**, 025502 (2006).

76. J. Miller, T. Boehly, A. Melchior, D. Meyerhofer, P. Celliers, J. Eggert, D. Hicks, C. Sorce, J. Oertel, P. Emmel, Streaked optical pyrometer system for laser-driven shock-wave experiments on omega. *Rev. Scient. Instrum.* **78**, 034903 (2007).

77. A. B. Belonoshko, S. Arapan, R. Martonak, A. Rosengren, MgO phase diagram from first principles in a wide pressure-temperature range. *Phys. Rev. B* **81**, 054110 (2010).

78. H. Abu-Farsakh, I. Al-Qasir, A. Qteish, Fundamental properties and phase stability of B1 and B2 phases of MgO over a wide range of pressures and temperatures: A first-principles study. *Comput. Mater. Sci.* **154**, 159–168 (2018).

79. W. Nellis, A. Mitchell, D. Young, Equation-of-state measurements for aluminum, copper, and tantalum in the pressure range 80–440 GPa (0.8–4.4 Mbar). *J. Appl. Phys.* **93**, 304–310 (2003).

80. Z. Wu, R. M. Wentzcovitch, K. Umemoto, B. Li, K. Hirose, J.-C. Zheng, Pressure-volume-temperature relations in MgO: An ultrahigh pressure-temperature scale for planetary sciences applications. *J. Geophys. Res.* **113**, (2008).

81. R. Musella, S. Mazevet, F. Guyot, Physical properties of MgO at deep planetary conditions. *Phys. Rev. B* **99**, 064110 (2019).

82. A. B. Belonoshko, L. S. Dubrovinsky, Molecular dynamics of NaCl (B1 and B2) and MgO (B1) melting: Two-phase simulation. *Am. Mineral.* **81**, 303–316 (1996).

83. N. De Koker, L. Stixrude, Self-consistent thermodynamic description of silicate liquids, with application to shock melting of MgO periclase and MgSiO3perovskite. *Geophys. J. Int.* **178**, 162–179 (2009).

84. A. Zerr, R. Boehler, Constraints on the melting temperature of the lower mantle from high-pressure experiments on MgO and magnesioüstite. *Nature* **371**, 506–508 (1994).

85. E. Ohtani, Melting temperature distribution and fractionation in the lower mantle. *Phys. Earth Planet. Inter.* **33**, 12–25 (1983).

86. A. Strachan, T. Çağin, W. A. Goddard III, Phase diagram of MgO from density-functional theory and molecular-dynamics simulations. *Phys. Rev. B* **60**, 15084–15093 (1999).

87. L. Zhang, Y. Fei, Melting behavior of (Mg, Fe)O solid solutions at high pressure. *Geophys. Res. Lett.* **35**, L13302 (2008).

88. O. Fat'yanov, P. Asimow, T. Ahrens, Thermodynamically complete equation of state of MgO from true radiative shock temperature measurements on samples preheated to 1850 K. *Phys. Rev. B* **97**, 024106 (2018).

89. D. Valencia, R. J. O'Connell, D. Sasselov, Internal structure of massive terrestrial planets. *Icarus* **181**, 545–554 (2006).

90. B. Svendsen, T. J. Ahrens, Shock-induced temperatures of MgO. *Geophys. J. R. Astr. Soc.* **91**, 667–691 (1987).

91. Y. Luo, S. Xiang, J. Li, J. Wu, L. Liu, J. Li, Y. Xian, R. Wu, Equation of state of MgO up to 345 GPa and 8500 K. *Phys. Rev. B* **107**, 134116 (2023).

92. D. Alfe, M. Alfredsson, J. Brodholt, M. Gillan, M. Towler, R. Needs, Quantum Monte Carlo calculations of the structural properties and the B1-B2 phase transition of MgO. *Phys. Rev. B* **72**, 014114 (2005).
